# Supplementary material for: Production of fatty acid-derived oleochemicals and biofuels by synthetic yeast cell factories
Source: Nat Commun. 2016 May 25;7:11709. doi: 10.1038/ncomms11709 (PMC4894961; doi:10.1038/ncomms11709)
Supplement: Supplementary Information — Supplementary Figures 1-12, Supplementary Tables 1-5 and Supplementary References. [file ncomms11709-s1.pdf]

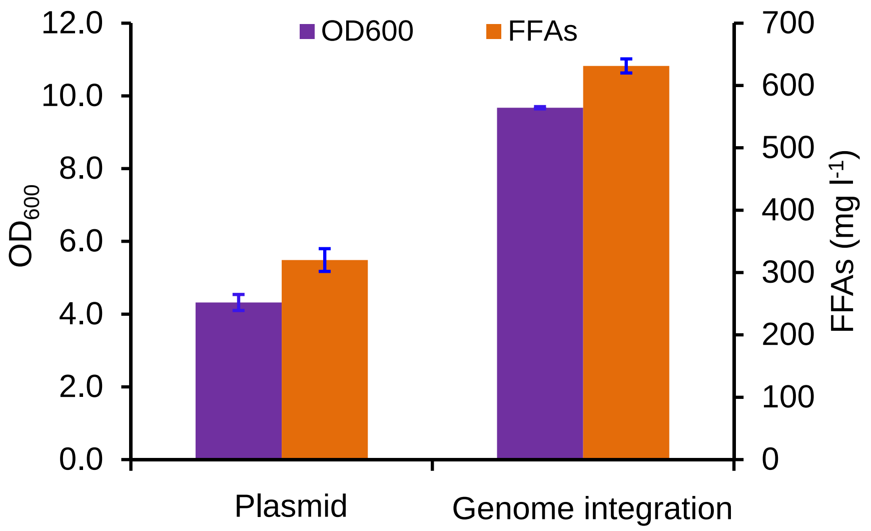

Supplementary Fig. 1. Genome integrated acetyl-CoA pathway had higher biomass and FFA titer compare to plasmid pathway. The plasmid pCoA4 transformed to YJZ08 and the same pathway was integrated to the YJZ08 genome. All data represent the mean ± s.d. of biological triplicates.

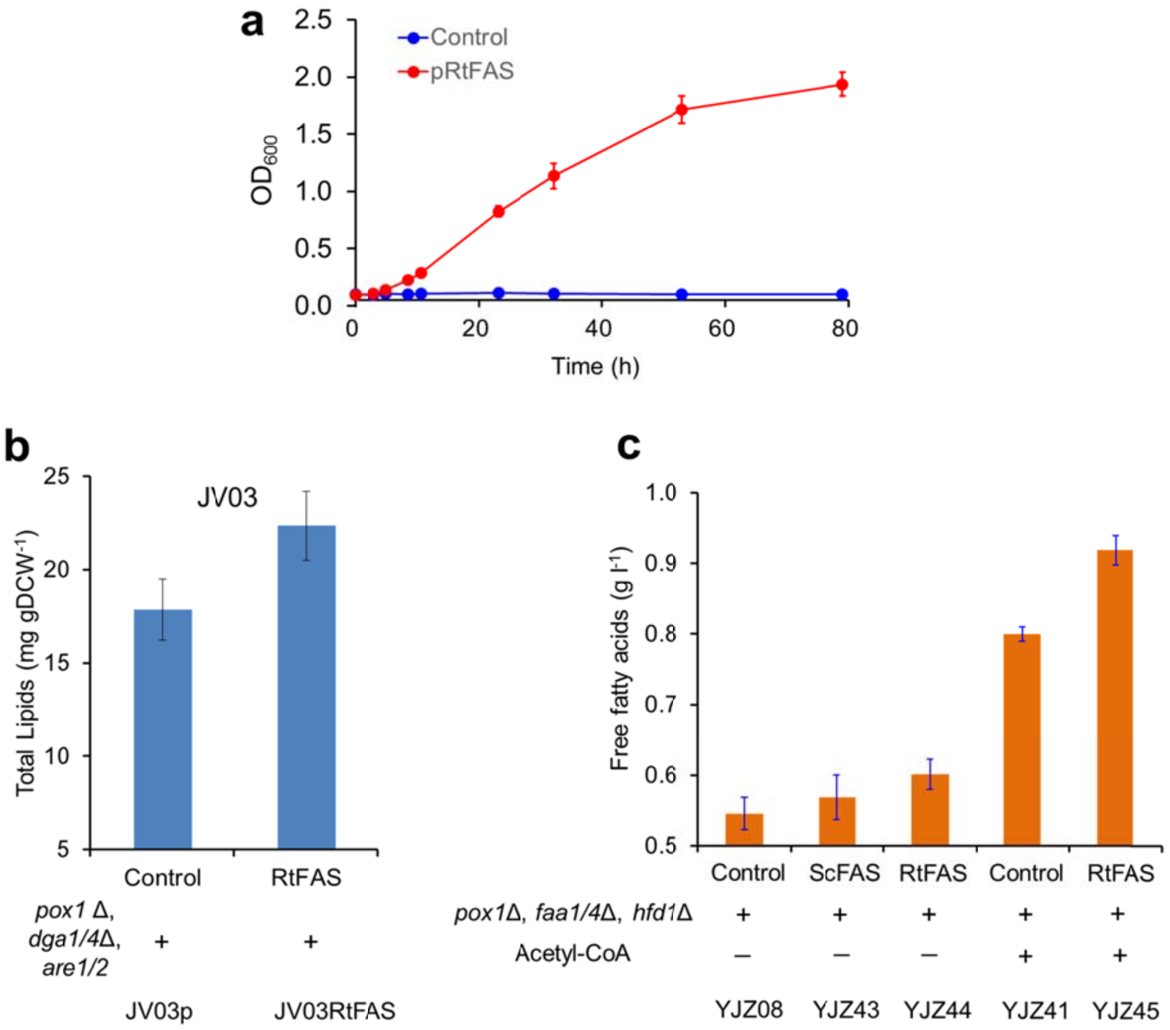

Supplementary Fig. 2. Expression of *R. toruloides* fatty acid synthase (RtFAS) increased the total lipid and FFA production. (A) RtFAS complemented the endogenous *FAS* deletion in *S. cerevisiae* PWY12 (*Δfas1::LEU2, Δfas2::HIS3*). (B) Expression of RtFAS increased the total lipid content in a JV03 background. (C) Expression of RtFAS was more efficient than overexpression of endogenous ScFAS in terms of increasing FFA production. All data represent the mean±s.d. of biological triplicates.

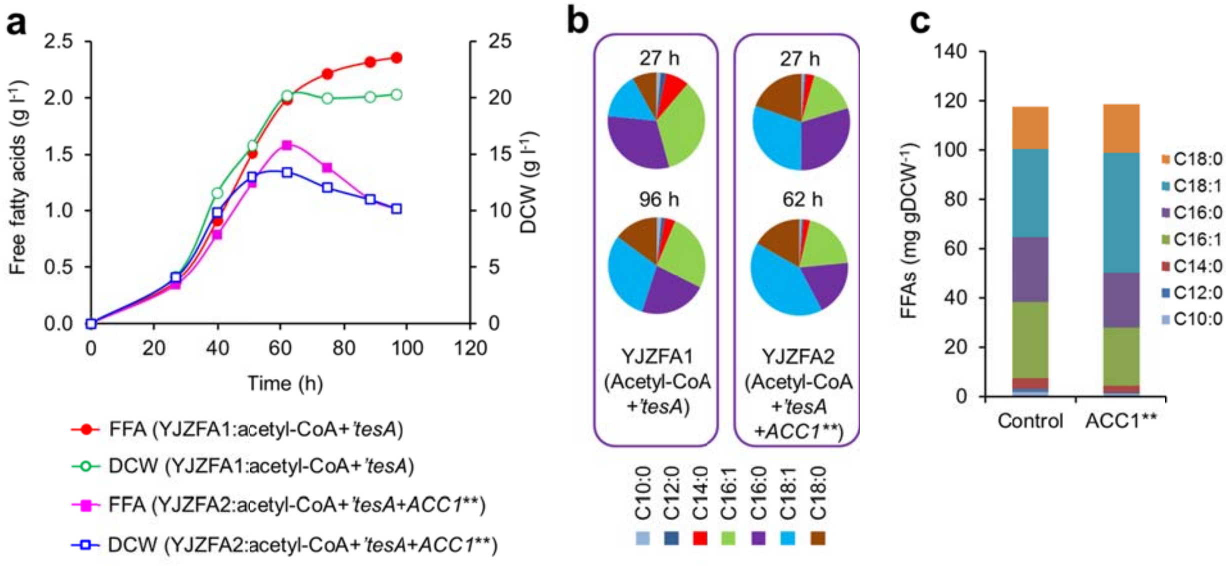

Supplementary Fig. 3. Effect of acetyl-CoA carboxylase mutant (Acc1^{S1157A,S659A}, Acc1**) on FFA production. (**a**) Fed batch fermentation of strains YJZFA1 and YJZFA2. Time courses of FFA titers (filled symbols) and cell mass (open symbols) are shown. YJZFA1 is a prototrophic strain harboring the ACL based pathway in plasmid pFab1 and YJZFA2 additionally expressesed *ACC1***. Here, 300 g l^{-1} glucose was fed and 2 M KOH was used for pH control other than described in Methods. (**b**) FFA profiles of the strain YJZFA1 and YJZFA2 at the time point where feeding was initiated and at the highest titer. (C) The maximal biomass specific free fatty acid titer.

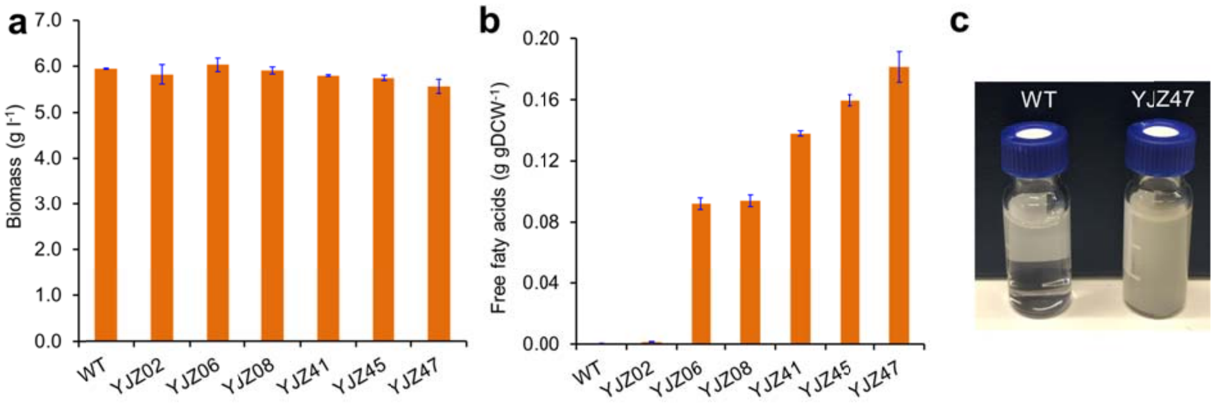

Supplementary Fig. 4 Comparison the biomass titers and specific FFA titers among recombinant *S. cerevisiae* variants. (**a**) The final biomass titers. (**b**) The specific FFA titers. (**c**) Comparison of cell culture between wild-type strain and final strain YJZ47. The cell cultures were centrifuged and the cell pellets were removed. The engineered strains were cultivated in shake flasks containing 15 mL optimized minimal media for 72 h at 200 rpm, 30 °C. All data represent the mean ± s.d. of biological triplicates.

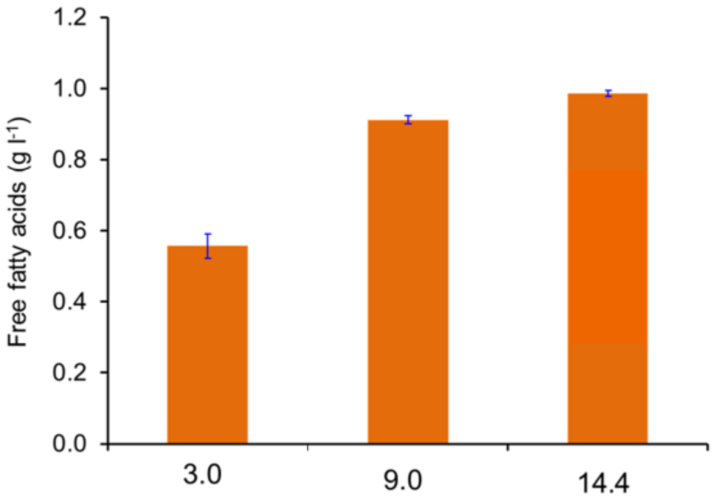

Supplementary Fig. 5 the effect of KH_2PO_4 level on FFA production. The other media components were 5 g l^{-1} $(NH_4)_2SO_4$, 0.5 g l^{-1} $MgSO_4 \bullet 7H_2O$, 30 g l^{-1} glucose, trace metal and vitamin solutions[1] supplemented with 40 mg l^{-1} histidine and/or 60 mg l^{-1} uracil. The engineered strain YJZ47were cultivated in shake flasks containing 15 mL media for 72 h at 200 rpm, 30 °C. All data represent the mean$\pm$s.d. of biological triplicates.

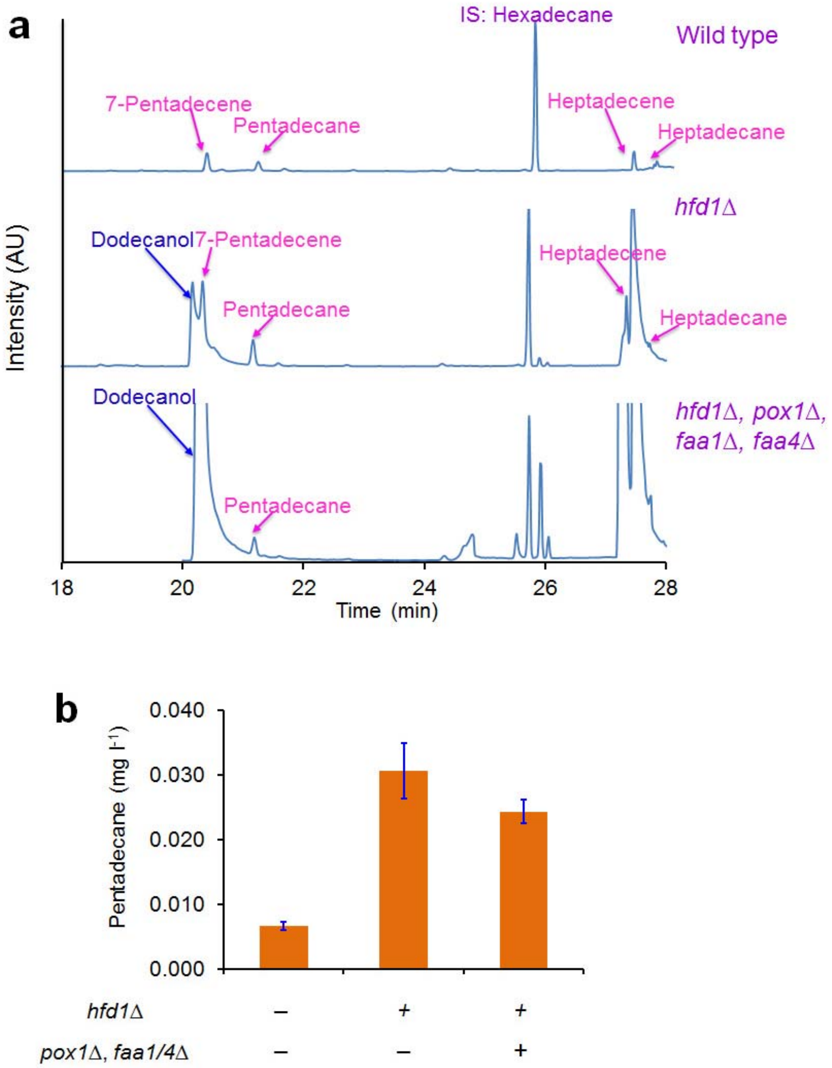

Supplementary Fig. 6. The ADO catalyzed step is rate limiting for alkane production, precursors are in sufficient supply. (a) GC chromatograms of extracted metabolites from different background strains harboring pAlkane16. *HFD1* deletion (middle chromatogram) increased the production of alkanes and fatty alcohols compared with the wild-type background (top chromatogram), which might be attributed to the blockage of fatty aldehyde dehydrogenation to fatty acids. Further increasing fatty acid supply (*hfd1*Δ *pox1*Δ *faa1*Δ *faa4*Δ) increased the amount of fatty alcohols, whose peaks covered the alkane peaks (bottom chromatogram). (b) Pentadecane production in the corresponding strains. The data represent the mean±s.d. of biological triplicates. As the peaks of 7-pentadecene and 8-heptadecene were covered by the fatty alcohols in the fatty acid overproducing strain, it became impossible to reliably quantify the alkanes. We thus compared the pentadecane titers, which indicated that a higher fatty acid supply did not increase the alkane but fatty alcohol production instead. These results indicated that the ADO is a limiting step and fatty acids might inhibit ADO activity, as the ADO has been shown to have fatty acid binding activity[2].

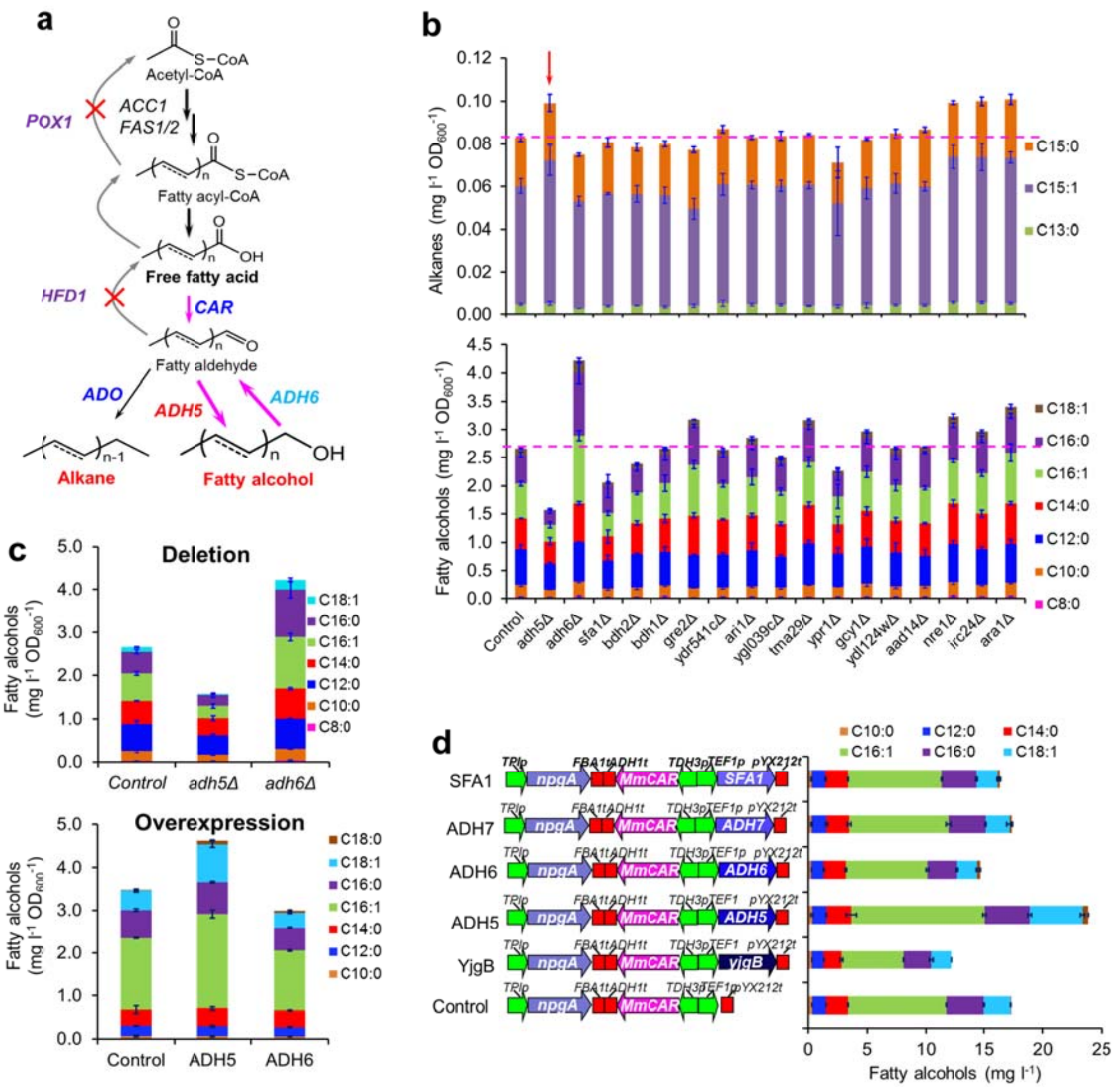

Supplementary Fig. 7 Engineering expression of alcohol dehydrogenase/aldehyde reductase genes for production of alkanes or fatty alcohols, (**a**) Schematic illustration of the engineered metabolic pathways of fatty alcohol biosynthesis. (**b**) The effect of single gene deletions on production of alkanes (top) and fatty alcohols (bottom). The targeted genes, encoding an alcohol dehydrogenase or aldehyde reductase, were selected based on the catalytic efficiency toward aldehyde reduction (Supplementary Table 1). And they were deleted in the YJZ03 background, and plasmid pAlkane16 was introduced for alkane production. Here, *ADH5* deletion showed increased alkane production and decreased fatty alcohol accumulation, and was considered as the first target for improving alkane production. (**c**) Opposite roles of alcohol dehydrogenases Adh5 and Adh6 in fatty alcohol biosynthesis. The effect of *ADH5* or *ADH6* deletion on fatty alcohol accumulation in strain YJZ03 (*hfd1*Δ and *pox1*Δ) harboring the plasmid pAlkane16 (top panel). The effect of *ADH5* or *ADH6* overexpression on fatty alcohol production in strain YJZ01 (*hfd1*Δ) harboring PAOH0 (bottom panel). (**d**) The amount of fatty alcohol produced with overexpression of genes encoding different alcohol dehydrogenase or aldehyde reductase in YJZ01. The data represent the mean ± s.d. of three independent clones.

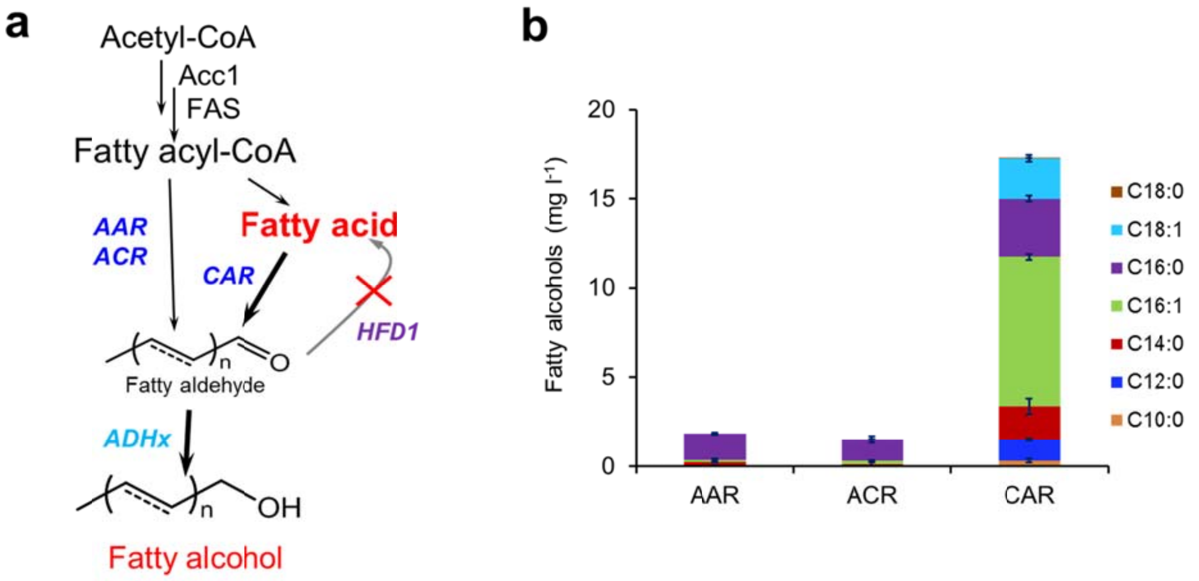

Supplementary Fig. 8 Comparison of fatty alcohol production with the expression of fatty acyl-CoA reductase ACR from *Acinetobacter baylyi*, fatty acyl-ACP/CoA reductase AAR from *Synechococcus elongatus* or carboxylic acid reductase CAR from *Mycobacterium marinum* (with its cofactor encoding gene *npgA* from *Aspergillus nidulans*). (**a**) Schematic illustration of the engineered metabolic pathways for fatty alcohol production. (**b**) CAR overexpression resulted in a much higher fatty alcohol level compared with overexpression of AAR and ACR. All data are presented as the mean±s.d. of biological triplicates.

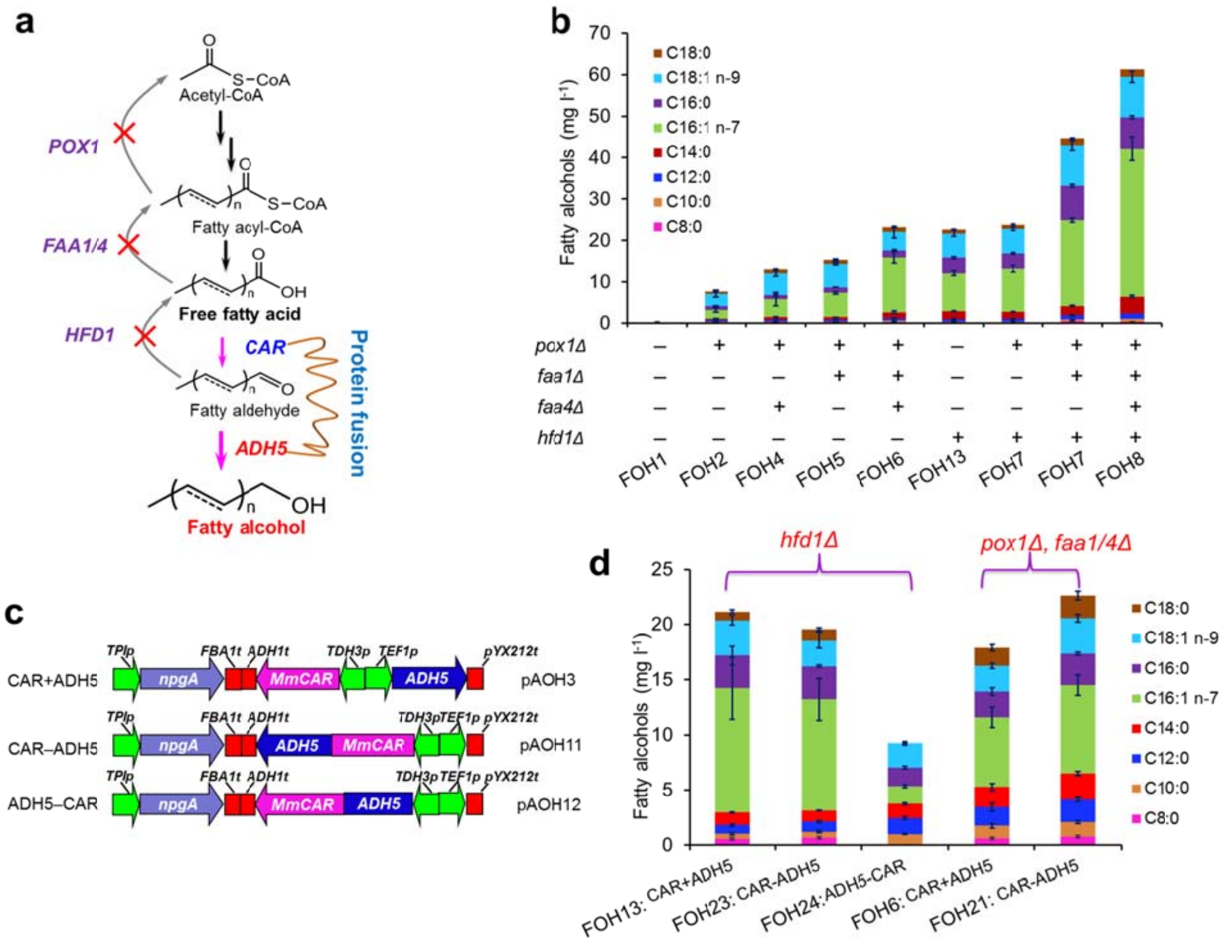

Supplementary Fig. 9 Engineered fatty alcohol production by blocking the reverse reactions and enzyme fusion. (**a**) Schematic representation of the engineered fatty alcohol biosynthetic pathways. (**b**) Fatty alcohol production from the engineered strains with deletion of *POX1*, *FAA1*, *FAA4* and *HFD1*. (**c**) Schematic representation of gene fusion constructs for fatty alcohol biosynthesis, CAR+ADH5 represents non-fusion expression of *CAR* and *ADH5* (plasmid pAOH3); CAR-ADH5 represents the fusion expression of *CAR-ADH5* with the CAR at N-terminus (plasmid pAOH11), and ADH5-CAR represents the fusion expression of *ADH5-CAR* with the Adh5 at the N-terminus (plasmid pAOH12). The fusion enzymes encoding genes were constructed by inserting a widely used GGGS linker encoding sequence "GGT GGT GGT TCT" between the two corresponding genes. (**d**) The amount of fatty alcohol produced by the fatty acid overproducing strain (YJZ06, *Δpox1; Δfaa1; Δfaa4*) and *HFD1* deletion strain (YJZ01) harboring different plasmids represented in c. The strain variants were cultivated in shake flasks for 72 h, at 30°C, 200 rpm. The data represent the mean ± s. d. of three independent clones.

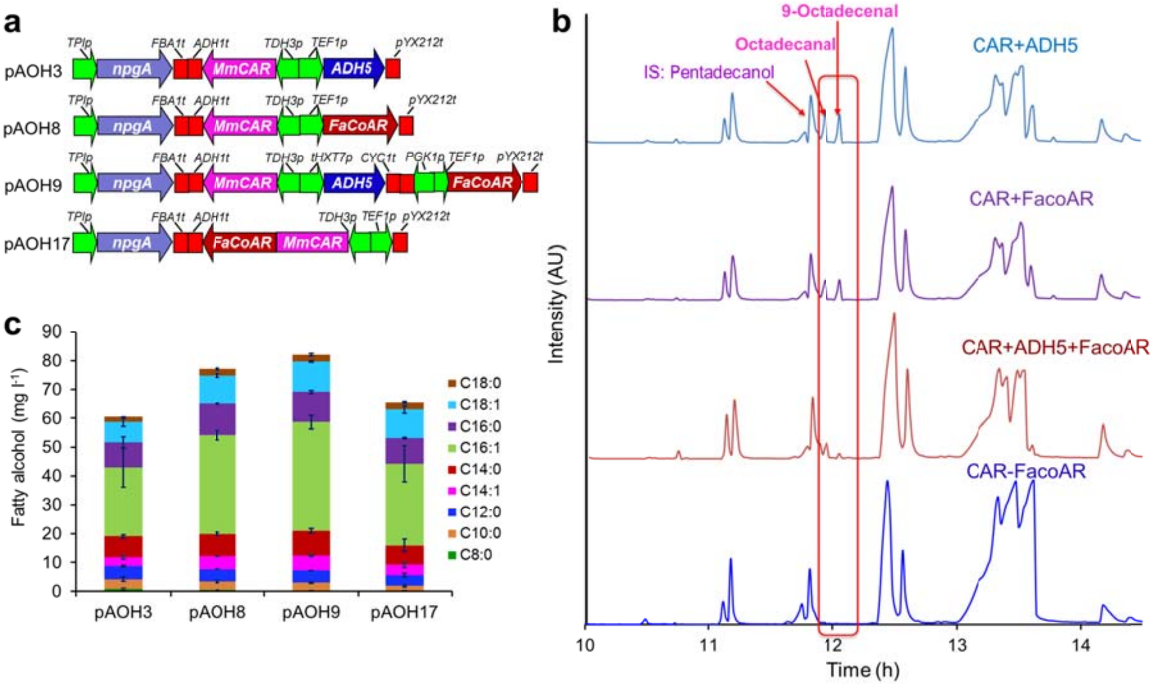

Supplementary Fig. 10 Overexpression of *FaCoAR* and *ADH5* increased long chain fatty alcohol production and decreased C18 fatty aldehyde accumulation. (**a**) Schematic representation of gene arrangement of the metabolic pathway for fatty alcohol biosynthesis. All these pathways were assembled on a pYX212 vector and then transformed into YJZ08 (*hfd1Δ pox1Δ faa1Δ faa4Δ*). (**b**) GC chromatograms of the extracts from the strains containing the corresponding pathways as shown in a. *FacoAR* expression decreased the accumulation of C18 fatty aldehyde octadecanal and 9-octadecenal. The fusion of CAR and FaCoAR avoided accumulation of octadecanal and 9-octadecenal completely, though the total fatty alcohol titer decreased by 15.2% as shown in c. (**c**) Titer of fatty alcohols from the strains harboring the corresponding plasmids. The data represent the mean $\pm$ s.d. of three independent clones.

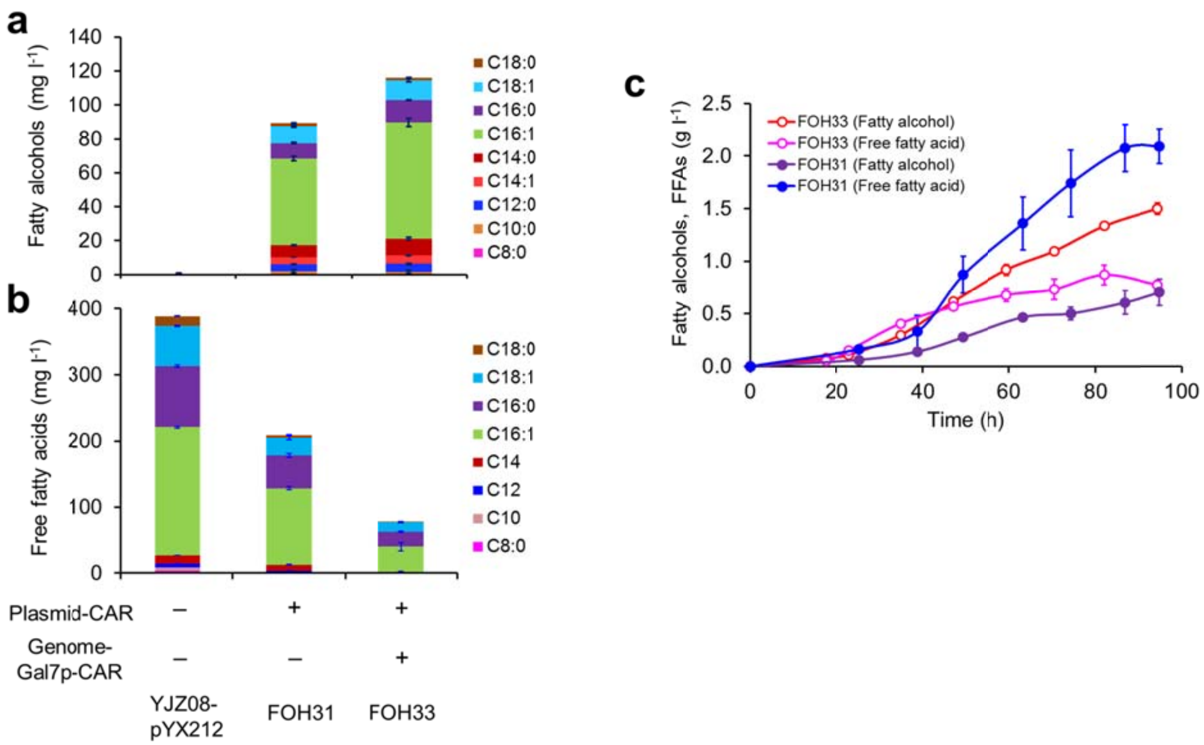

Supplementary Fig. 11. Enhancing fatty acid reduction for fatty alcohol production. (**a**) Fatty alcohol titer in shake flasks. (**b**) FFA titer from the strains harboring the corresponding pathways in shake flasks. The strain variants were cultivated in minimal media for 72 h, at 30°C, 200 rpm. The data represent the mean ± s.d. of three independent clones. (**c**) The accumulation of fatty alcohols and FFAs from FOH11 and FOH33 in fed-batch fermentation.

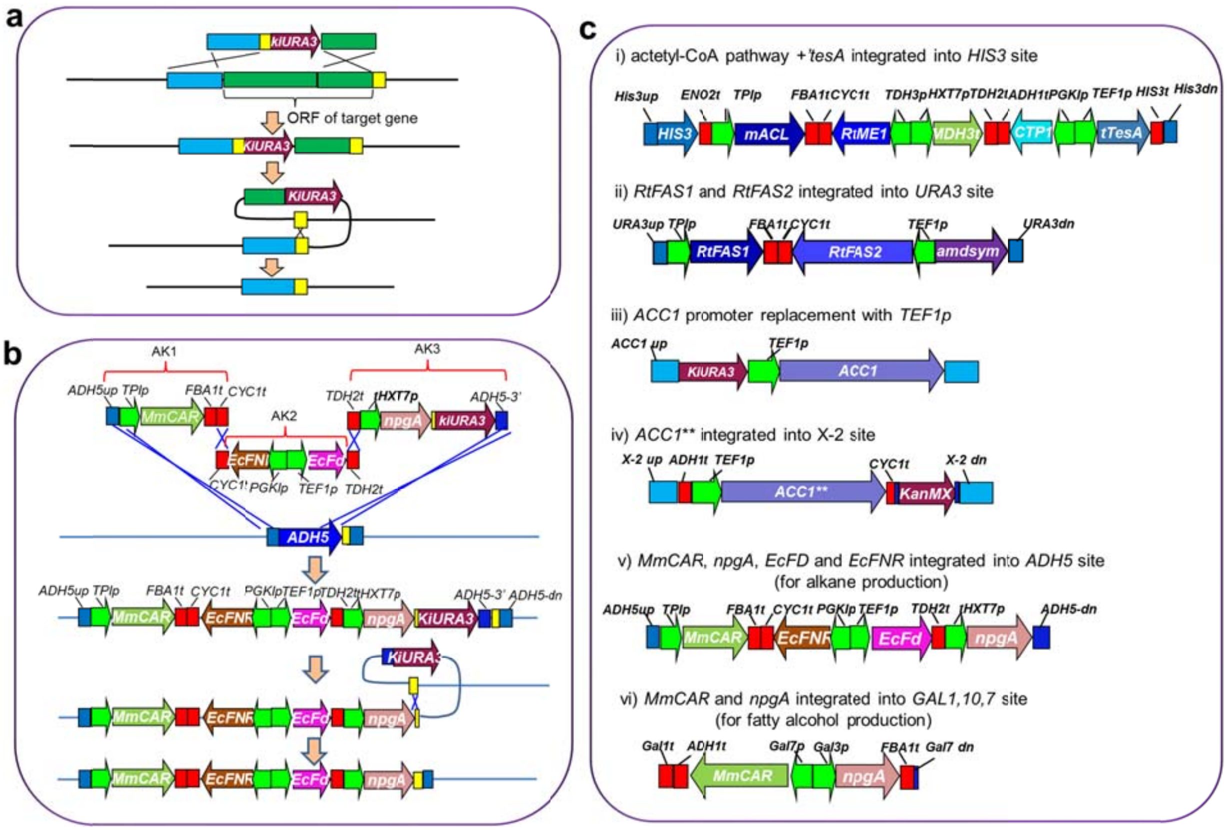

Supplementary Fig. 12 Schematic illustration of genome engineering strategy. (**a**) The seamless gene deletion strategy. (**b**) Modular pathway integration strategy. (**c**) The genetic arrangement of genome-integrated pathways.

Supplementary Table 1. *S. cerevisiae* (putative) alcohol dehydrogenases and aldehyde reductases[a]

Enzyme	Descriptions	localization
**Medium to long-chain alcohol dehydrogenase (alcohol formation)**		
ADH3 (YMR083W)	Involved in shuttling of mitochondrial NADH to the cytosol under anaerobic conditions and ethanol production[4].	Mitochondria
ADH4 (YGL256W)	Adh4 is seemingly not expressed in laboratory *S. cerevisiae* strains.	Cytoplasm
**ADH5 (YBR145W)**	Overexpression of *ADH5* increased isobutanol production in *S. cerevisiae*[5].	Cytoplasm
**ADH6 (YMR318C)**	NADPH-dependent aldehyde reductase. k_{cat}/K_m is 1-3 × 10^5 mM^{-1} min^{-1} (Ref. 6); *ADH6* deletion decreased the reduction of vanillin to vanillyl alcohol[7].	Cytoplasm
ADH7 (YCR105W)	Involved in fusel alcohol synthesis or in aldehyde tolerance[8]. Absent in CEN.PK strains.	Cytoplasm
**SFA1 (YDL168W)**	Reduction of long chain and complex aldehydes to corresponding alcohols[9].	Cytoplasm/ Mitochondria
XYL2 (YLR070C)	Xylitol dehydrogenase that converts xylitol to D-xylulose[10].	Unknown
**BDH1 (YAL061w)**	NAD-dependent butanediol dehydrogenase, catalyzes reduction of acetoin to 2,3-butanediol[11,12].	Cytoplasm
**BDH2 (Yal060w)**	Homolog of *BDH1*	Cytoplasm
**GRE2 (YOL151W)**	Reduction activity toward complex aldehydes and ketones[13].	Cytoplasm
**ARI1 (YGL157w)**	Reduction activity toward furan aldehydes[14] and high activity (k_{cat}/K_m=550 min^{-1} mM^{-1}) toward phenylacetaldehyde[15].	Cytoplasm/ Nucleus
**YGL039w**	Reduction activity toward phenylacetaldehyde[15], short chain fatty aldehydes with a NADH preference[16].	Cytoplasm
**YDR541c**	Reduction activity toward fatty aldehydes (up to C8) and benzyl aldehydes with a NADPH preference[16].	Unknown
YBR159w	3-ketoreductase of the microsomal fatty acid elongase[17].	Endoplasmic reticulum
**TMA29 (YMR226c)**	Reduction activity toward aromatic α-ketoesters and aliphatic ketones[18].	Cytoplasm/ Nucleus
**NRE1 (YIR035c)**	Short chain alcohol dehydrogenase[19].	Cytosol
**IRC24 (YIR036c)**	Reduction activity toward benzyl (k_{cat}/K_m=44 min^{-1} mM^{-1}) and 1-phenyl-1,2-	Cytoplasm

	propanedione (k_{cat}/K_m=3 000 min^{-1} mM^{-1}) [20].	
AYR1 (YIL124w)	1-Acyldihydroxyacetone-phosphate reductase[21]	Lipid particles
YKL107w	Putative short-chain dehydrogenase/reductase, proposed to be a palmitoylated membrane protein	Unknown
**AAD14 (YNL331c)**	Aryl alcohol dehydrogenase[22], combination of Aad3, 4, 10, 14-16 may be involved in biosynthesis of long-chain and complex alcohols[9]	Unknown
AAD3 (YCR107w)	Homolog of *AAD14*	Unknown
AAD4 (YDL243c)	Homolog of *AAD14*	Unknown
AAD10 (YJR155w)	Homolog of *AAD14*	Unknown
AAD16 (YFL057c)	Homolog of *AAD14*	Unknown
AAD15 (YOL165c)	Homolog of *AAD14*	Unknown
**Aldose reductase family**		
**YPR1 (YDR368w)**	Reduction activity toward diacetyl and ethyl acetoacetate[19]	Cytoplasm/ Nucleus
**GCY1 (YOR120w)**	High activity toward dl-glyceraldehyde (k_{cat}/K_m =556 min^{-1} mM^{-1}) and nitrobenzaldehyde (k_{cat}/K_m =546 min^{-1} mM^{-1}) [23,24]	Cytoplasm/ Nucleus
**YDL124w**	Reduction activity toward dl-Glyceraldehyde (k_{cat}/K_m =17 min^{-1} mM^{-1}) and nitrobenzaldehyde k_{cat}/K_m =110 min^{-1} mM^{-1}) but lower than Gcy1[23]	Cytoplasm/ Nucleus
YJR096w	Much lower reduction activity than Gcy1 and YDL124Wp[23]	Cytoplasm/ Nucleus
**ARA1 (YBR149w)**	Showed reduction activity toward diacetyl[25] and acetoin[12]	Cytoplasm
GRE3 (YHR104w)	Involved in reduction of aldoses such as d-xylose[10,26].	Cytoplasm/ Nucleus
**D-Hydroxyacid dehydrogenase family**		
GOR1 (YNL274c)	Glyoxylate reductase, showed activity toward glyoxylate and hydroxypyruvate[27]	Cytoplasm/ Mitochondria
YPL113c	Glyoxylate reductase, showed activity toward glyoxylate and hydroxypyruvate, but much lower compared with Gcor1[27]	Unknown
YGL185c	Glyoxylate reductase, showed activity toward glyoxylate and hydroxypyruvate, but much lower compared with Gcor1[27]	Cytoplasm

FDH1 (YOR388c)	NAD^+-dependent formate dehydrogenase[28]	Cytoplasm
FDH2 (YPL275w)	NAD^+-dependent formate dehydrogenase[28]	Cytoplasm

a, Genes selected for deletion are indicated in bold

Supplementary Table 2. Plasmids used in this study[a]

Plasmids	Genotype or characteristic	Resource
pYX212	2 µm, AmpR, *URA3*, TPIp, pYX212t	R&D systems
pCoA1	pYX212-(*TPIp*-***RtACL***-*FBA1t*)+(*TDH3p*-***RtME***-*CYC1t*)	This study
pCoA2	pYX212-(*TPIp*-***RtACL***-*FBA1t*)+(*TDH3p*-***LsME***-*CYC1t*)	This study
pCoA3	pYX212-(*TPIp*-***RtACL***-*FBA1t*)+(*TDH3p*-***RtME***-*CYC1t*)+(tHXT7p-***'MDH3***-*pYX212t*)	This study
pCoA4	pYX212-(*TPIp*-***RtACL***-*FBA1t*)+(*TDH3p*-***RtME***-*CYC1t*)+(tHXT7p-***'MDH3***-*TDH2t)*+(*PGK1p*-***CTP1***-*ADH1t*)	This study
pFab1	pYX212-(*TPIp*-***RtACL***-*FBA1t*)+(*TDH3p*-***RtME***-*CYC1t*)+(tHXT7p-***'MDH3***-*TDH2t)*+(*PGK1p*-***CTP1***-*ADH1t*)+(*TEF1p*-***'tesA***-*pYX212t*)	This study
pFab3	pYX212-(*TPIp*-***RtACL***-*FBA1t*)+(*TDH3p*-***RtME***-*CYC1t*)+(tHXT7p-***'MDH3***-*TDH2t)*+(*PGK1p*-***CTP1***-*ADH1t*)+(*TEF1p*-***fadM***-*pYX212t*)	This study
pRtFAS	pYX212-(*TPIp*-***RtFAS1***-*FBA1t*)+ (*TEF1p*-***RtFAS2***-*CYC1t*)	This study
pAOH0	pYX212-(*TPIp*-***npgA***-*FBA1t*)+(*TDH3p*-***MmCAR***-*ADH1t*)	This study
pAOH1	pYX212-(*TPIp*-***npgA***-*FBA1t*)+(*TDH3p*-***MmCAR***-*ADH1t*)+(*TEF1p*-***yjgB***-*pYX212t*)	This study
pAOH3	pYX212-(*TPIp*-***npgA***-*FBA1t*)+(*TDH3p*-***MmCAR***-*ADH1t*)+(*TEF1p*-***ADH5***-*pYX212t*)	This study
pAOH4	pYX212-(*TPIp*-***npgA***-*FBA1t*)+(*TDH3p*-***MmCAR***-*ADH1t*)+(*TEF1p*-***ADH6***-*pYX212t*)	This study
pAOH5	pYX212-(*TPIp*-***npgA***-*FBA1t*)+(*TDH3p*-***MmCAR***-*ADH1t*)+(*TEF1p*-***ADH7***-*pYX212t*)	This study
pAOH6	pYX212-(*TPIp*-***npgA***-*FBA1t*)+(*TDH3p*-***MmCAR***-*ADH1t*)+(*TEF1p*-***SFA1***-*pYX212t*)	This study
pAOH8	pYX212-(*TPIp*-***npgA***-*FBA1t*)+(*TDH3p*-***MmCAR***-*ADH1t*)+(*TEF1p*-***FacoAR***-*pYX212t*)	This study
pAOH9	pYX212-(*TPIp*-***npgA***-*FBA1t*)+(*TDH3p*-***MmCAR***-*ADH1t*)+(*tHXT7p*-***ADH5***-*CYC1t*)+(*TEF1p*-***FacoAR-***pYX212t*)	This study
pAOH11	pYX212-(*TPIp*-***npgA***-*FBA1t*)+(*TDH3p*-***MmCAR-ADH5***-*ADH1t*)	This study
pAOH12	pYX212-(*TPIp*-***npgA***-*FBA1t*)+(*TDH3p*-***ADH5-MmCAR***-*ADH1t*)	This study
pAOH17	pYX212-(*TPIp*-***npgA***-*FBA1t*)+(*TDH3p*-***MmCAR-FacoAR***-*ADH1t*)	This study

pAlkane7	pYX212-(*TPIp*-***SeAAR***-*FBA1t*)+(PGK1p-***EcFNR***-*CYC1t*)+(*TEF1p*-***EcFD***-*TDH2t*)+(*TDH3p*-***SeADO***-*ADH1t*)	29
pAlkane16	pYX212-(*TPIp*-***MmCAR***-*FBA1t*)+(PGK1p-***EcFNR***-*CYC1t*)+(*TEF1p*-***EcFD***-*TDH2t*)+(*TDH3p*-***SeADO***-*ADH1t*)+(tHXT7p-***npgA***-*pYX212t*)	This study
pAlkane65	pYX212-(*UAS-TDH3p*-***SeADO***-*pYX212t*)	This study
pAlkane67	pYX212-(*UAS-TDH3p*-***SeADO***-*pYX212t)*	This study
pAlkane68	pYX212-(*GAL7p*-***NpADO***-*CYC1t*)+(*UAS-TDH3p*-**SeADO**-*pYX212t*)	This study

a, Expressed genes are indicated in bold

b, per1 means the peroxisome targeting peptide 1 encoding sequence: GGTGGTGGTTCTTCTAAACTA and per2 means peroxisome targeting peptide 1 encoding sequence: GGTGGTGGTTCTGCCGCTGTAAAACTATCGCAGGCAAAATCTAAACTA

Supplementary Table 3 *S. cerevisiae* strains used in this study.

Strain	Genotype or characteristic	Resource
**Background strains/fatty acid producing strains**		
CEN.PK 113-11C	*MATa MAL2-8c SUC2 his3Δ1 ura3-52*	Kötter, University of Frankfurt, Germany
EY1673	*MATα his3Δ1 leu2Δ0 lys2Δ0 ura3Δ0 PEX3-mRFP+kanMX6*	[3]
YJZ01	*MATa MAL2-8c SUC2 his3Δ1 ura3-52 hfd1Δ*	[29]
YJZ02	*MATa MAL2-8c SUC2 his3Δ1 ura3-52 pox1Δ*	This study
YJZ03	*MATa MAL2-8c SUC2 his3Δ1 ura3-52 hfd1Δ pox1Δ*	This study
YJZ04	*MATa MAL2-8c SUC2 his3Δ1 ura3-52 pox1Δ faa4Δ*	This study
YJZ05	*MATa MAL2-8c SUC2 his3Δ1 ura3-52 pox1Δ faa1Δ*	This study
YJZ06	*MATa MAL2-8c SUC2 his3Δ1 ura3-52 pox1Δ faa1Δ faa4Δ*	This study
YJZ07	*MATa MAL2-8c SUC2 his3Δ1 ura3-52 hfd1Δ pox1Δ faa1Δ*	This study
YJZ08	*MATa MAL2-8c SUC2 his3Δ1 ura3-52 hfd1Δ pox1Δ faa1Δ faa4Δ*	This study
JV03	*MATa MAL2-8c SUC2 ura3-52 HIS3 are1Δ dga1Δ are2Δ lro1Δ pox1Δ*	[30]
JV03RtFAS	*MATaMAL2-8c SUC2 ura3-52 HIS3 are1Δ dga1Δ are2Δ lro1Δ pox1Δ* pRtFAS	This study
RWB837	*MATa pdc1Δ(-6,-2)::loxP pdc5Δ(-6,-2)::loxP pdc6Δ(-6,-2)::loxP ura3-52*	[31]
IMI076	*MATa pdc1Δ(-6,-2)::loxP pdc5Δ(-6,-2)::loxP pdc6Δ(-6,-2)::loxP ura3-52 MTH1-ΔT*	[31]
FA0	*MATa MAL2-8c SUC2 his3Δ1 ura3-52 hfd1Δ pox1Δ faa1Δ faa4Δ pYX212*	This study
FA1	*MATa MAL2-8c SUC2 his3Δ1 ura3-52 hfd1Δ pox1Δ faa1Δ faa4Δ pCoA1*	This study
FA2	*MATa MAL2-8c SUC2 his3Δ1 ura3-52 hfd1Δ pox1Δ faa1Δ faa4Δ pCoA2*	This study
FA3	*MATa MAL2-8c SUC2 his3Δ1 ura3-52 hfd1Δ pox1Δ faa1Δ faa4Δ pCoA3*	This study
FA4	*MATa MAL2-8c SUC2 his3Δ1 ura3-52 hfd1Δ pox1Δ faa1Δ faa4Δ pCoA4*	This study
YJZFA1	*MATa MAL2-8c SUC2 his3Δ1 ura3-52 hfd1Δ pox1Δ faa1Δ faa4Δ* pFab1, p413::HIS3	This study
YJZFA2	*MATa MAL2-8c SUC2 his3Δ1 ura3-52 hfd1Δ pox1Δ faa1Δ faa4Δ X2::*(*TEF1p-**ACC1*****-*CYC1t*) pFab1 p413::HIS3	This study
YJZ13	*MATa MAL2-8c SUC2 ura3-52 hfd1Δ pox1Δ faa1Δ faa4Δ his3Δ::HIS3*+(*TEF1p-**'tesA**-HIS3t*)	This study
YJZ39	*MATa MAL2-8c SUC2 ura3-52 hfd1Δ pox1Δ faa1Δ faa4Δ his3Δ::HIS3*+(*TPIp-**RtACL**-FBA1t*)+(*TDH3p-**RtME**-CYC1t*)+(tHXT7p-*'**MDH3**-TDH2t*)+(*PGK1p-**CTP1**-ADH1t*)+(*TEF1p-**'tesA**-HIS3t*)	This study

YJZ41	*MATa; MAL2-8c SUC2 ura3-52 hfd1Δ pox1Δ faaΔ1 faa4Δ his3Δ::HIS3*+(*TPIp-**MmACL**-FBA1t*)+(*TDH3p-**RtME**-CYC1t*)+(tHXT7p-***'MDH3**-TDH2t)*+(*PGK1p-**CTP1**-ADH1t*)+(*TEF1p-**'tesA**-HIS3t*)	This study
YJZ42	*MATa MAL2-8c SUC2 ura3-52 hfd1Δ pox1Δ faa1Δ faa4Δ his3Δ::HIS3*+(*TPIp-**HsACL**-FBA1t*)+(*TDH3p-**RtME**-CYC1t*)+(tHXT7p-***'MDH3**-TDH2t)*+(*PGK1p-**CTP1**-ADH1t*)+(*TEF1p-**'tesA**-HIS3t*)	This study
YJZ43	*MATa MAL2-8c SUC2 his3Δ1 ura3-52 hfd1Δ pox1Δ faa1Δ faa4Δ ura3Δ::*(*TPIp-**ScFAS1**-FBA1t*)+ (*TEF1p-**ScFAS2**-CYC1t*)*+amdSym*	This study
YJZ44	*MATa MAL2-8c SUC2 his3Δ1 ura3-52 hfd1Δ pox1Δ faa1Δ faa4Δ ura3Δ::*(*TPIp-**RtFAS1**-FBA1t*)+ (*TEF1p-**RtFAS2**-CYC1t*)*+amdSym*	This study
YJZ45	*MATa MAL2-8c SUC2 ura3-52 hfd1Δ pox1Δ faa1Δ faa4Δ his3Δ::HIS3*+(*TPIp-**MmACL**-FBA1t*)+(*TDH3p-**RtME**-CYC1t*)+(tHXT7p-***'MDH3**-TDH2t)*+(*PGK1p-**CTP1**-ADH1t*)+(*TEF1p-**'tesA**-HIS3t*) *ura3Δ::*(*TPIp-**RtFAS1**-FBA1t*)+ (*TEF1p-**RtFAS2**-CYC1t*)*+amdSym*	This study
YJZ45U	*MATa MAL2-8c SUC2 ura3-52 hfd1Δ pox1Δ faa1Δ faa4Δ his3Δ::HIS3*+(*TPIp-**MmACL**-FBA1t*)+(*TDH3p-**RtME**-CYC1t*)+(tHXT7p-***'MDH3**-TDH2t)*+(*PGK1p-**CTP1**-ADH1t*)+(*TEF1p-**'tesA**-HIS3t*) *ura3Δ::*(*TPIp-**RtFAS1**-FBA1t*)+ (*TEF1p-**RtFAS2**-CYC1t*)*+amdSym p416::URA3*	This study
YJZ47	*MATa MAL2-8c SUC2 hfd1Δ pox1Δ faa1Δ faa4Δ his3Δ::HIS3*+(*TPIp-**MmACL**-FBA1t*)+(*TDH3p-**RtME**-CYC1t*)+(tHXT7p-***'MDH3**-TDH2t)*+(*PGK1p-**CTP1**-ADH1t*)+(*TEF1p-**'tesA**-HIS3t*) *ura3Δ::*(*TPIp-**RtFAS1**-FBA1t*)+ (*TEF1p-**RtFAS2**-CYC1t*)*+amdSym acc1::KlURA3+TEF1p+**ACC1***	This study
**Fatty alcohol producing strains**		
FOH1	CEN.PK 113-11C, pAOH3	This study
FOH2	*MATa MAL2-8cSUC2 his3Δ1 ura3-52 pox1Δ* pAOH3	This study
FOH3	*MATa MAL2-8c SUC2 his3Δ1 ura3-52 hfd1Δ pox1Δ* pAOH3	This study
FOH4	*MATa MAL2-8c SUC2 his3Δ1 ura3-52 pox1Δ faa4Δ* pAOH3	This study
FOH5	*MATa MAL2-8c SUC2 his3Δ1 ura3-52 pox1Δ faa1Δ* pAOH3	This study
FOH6	*MATa MAL2-8c SUC2 his3Δ1 ura3-52 pox1Δ faa1Δ faa4Δ* pAOH3	This study
FOH7	*MATa MAL2-8c SUC2 his3Δ1 ura3-52 hfd1Δ pox1Δ faa1Δ* pAOH3	This study
FOH8	*MATa MAL2-8c SUC2 his3Δ1 ura3-52 hfd1Δ pox1Δ faa1Δ faa4Δ* pAOH3	This study
FOH10	*MATa MAL2-8c SUC2 his3Δ1 ura3-52 hfd1Δ* pAOH0	This study
FOH11	*MATa MAL2-8c SUC2 his3Δ1 ura3-52 hfd1Δ* pAOH1	This study
FOH13	*MATa MAL2-8c SUC2 his3Δ1 ura3-52 hfd1Δ* pAOH3	This study

FOH14	*MATa MAL2-8c SUC2 his3Δ1 ura3-52 hfd1Δ* pAOH4	This study
FOH15	*MATa MAL2-8c SUC2 his3Δ1 ura3-52 hfd1Δ* pAOH5	This study
FOH16	*MATa MAL2-8c SUC2 his3Δ1 ura3-52 hfd1Δ* pAOH6	This study
FOH21	*MATa MAL2-8c SUC2 his3Δ1 ura3-52 pox1Δ faa1Δ faa4Δ* pAOH11	This study
FOH23	*MATa MAL2-8c SUC2 his3Δ1 ura3-52 hfd1Δ* pAOH11	This study
FOH24	*MATa MAL2-8c SUC2 his3Δ1 ura3-52 hfd1Δ* pAOH12	This study
FOH28	*MATa MAL2-8c SUC2 his3Δ1 ura3-52 hfd1Δ pox1Δ faa1Δ faa4Δ* pAOH8	This study
FOH29	*MATa MAL2-8c SUC2 his3Δ1 ura3-52 hfd1Δ pox1Δ faa1Δ faa4Δ* pAOH9	This study
FOH30	*MATa MAL2-8c SUC2 his3Δ1 ura3-52 hfd1Δ pox1Δ faa1Δ faa4Δ* pAOH17	This study
FOH31	*MATa MAL2-8c SUC2 ura3-52 hfd1Δ pox1Δ faa1Δ faa4Δ adh6Δ::kanMX*, pAOH9	This study
FOH33	*MATa MAL2-8c SUC2 ura3-52 hfd1Δ pox1Δ faa1Δ faa4Δ*, *adh6Δ::kanMX*, *gal80Δ*, *gal1/10/7Δ::*(*GAL7p-**MmCAR**-ADH1t*)+(GAL3p-***npgA***-*FBA1t*) pAOH9	This study
**Alkane producing strains**		
ZW31	*MATa MAL2-8c SUC2 his3Δ1 ura3-52 hfd1Δ pox1Δ adh5Δ*	This study
YJZ60	*MATa MAL2-8c SUC2 his3Δ1 ura3-52 hfd1Δ pox1Δ Gal80Δ:: SeFNR+SeFd adh5Δ::* (*TPIp-**MmCAR**-FBA1t*)+(PGK1p-***EcFNR***-*CYC1t*)+(*TEF1p-**EcFD**-TDH2t*)+(tHXT7p-***npgA***-*ADH5t*)	This study
A0	*MATa MAL2-8c SUC2 his3Δ1 ura3-52 hfd1Δ* pAlkane7 (previously named as KB19)	[29]
A1	*MATa MAL2-8c SUC2 his3Δ1 ura3-52 hfd1Δ* pAlkane06	This study
A2	*MATa MAL2-8c SUC2 his3Δ1 ura3-52 hfd1Δ pox1Δ* pAlkane06	This study
A3	*MATa MAL2-8c SUC2 his3Δ1 ura3-52 hfd1Δ pox1Δ adh5Δ* pAlkane06	This study
A5	*MATa MAL2-8c SUC2 his3Δ1 ura3-52 hfd1Δ pox1Δ Gal80Δ:: SeFNR+SeFd adh5Δ::* (*TPIp-**MmCAR**-FBA1t*)+(PGK1p-***EcFNR***-*CYC1t*)+(*TEF1p-**EcFD**-TDH2t*)+(tHXT7p-***npgA***-*ADH5t*) pAlkane67	This study
A6	*MATa MAL2-8c SUC2 his3Δ1 ura3-52 hfd1Δ pox1Δ Gal80Δ:: SeFNR+SeFd adh5Δ::* (*TPIp-**MmCAR**-FBA1t*)+(PGK1p-***EcFNR***-*CYC1t*)+(*TEF1p-**EcFD**-TDH2t*)+(tHXT7p-***npgA***-*ADH5t*) pAlkane68	This study

Supplementary Table 4. Primers used in this study

Primer No.	Name	Sequence (5'-3')
**Primers for seamless gene deletion of *POX1*, *FAA1* and *FAA4***		
p1	POX1(up)-F	GATTCCTTCAGTTCCACTTTTTGC
p2	POX1(up)-R	GAATTGAAACAAAAGTCGCAAAACAGAGGGTTCGAAGGAAAACAGGAAACCTCTACTC ACATATCGCAATACTAATTTATTAT
p3	KIURA3-F1	CTTCGAACCCTCTGTTTTGCGACTTTTGTTTCAATTCAACTAGTGTCGCCAAGTTTTAACG TGATTCTGGGTAGAAGATCG
p4	KIURA3-R2	GAGCCAATAGTTGTGGCTGCACAACTTTAGAGATCCATCGATAAGCTTGATATCG
p5	POX1(dw)-F	GATCTCTAAAGTTGTGCAGCCAC
p6	POX1(dw)-R	CGCATTAGCTGCACCACCTAAC
p7	FAA1(up)-F	CACCCACCCATCGCATATCAGG
p8	FAA1(up)-R	CTGAAAAAGTGCTTTAGTATGATGAGGCTTTCCTATCATGGAAATGTTGATCCATTACA TATTGTTGTCTTTTTTTGTC
p9	KIURA3-F2	GATAGGAAAGCCTCATCATACTAAAGCACTTTTTCAGTTTTTTGCTTTAGAACTGCTACC GTGATTCTGGGTAGAAGATCG
p10	KIURA3-R2	CAACATATTCGTTAGATCTGTAAACGGACTCTAATTTCCATCGATAAGCTTGATATCG
p11	FAA1(dw)-F	GAAATTAGAGTCCGTTTACAGATC
p12	FAA1(dw)-R	GTCAAAGAACACTATGCCTGCTAG
p13	FAA4(up)-F	GTCCCCATCAATTAAGAACCCTC
p14	FAA4(up)-R	GAAAATGAAACGTAGTGTTTATGAAGGGCAGGGGGGAAAGTAAAAAACTATGTCTTCC TTTACATTTTGATGCGTACTTCTTAG
p15	KIURA3-F3	CTTTCCCCCCTGCCCTTCATAAACACTACGTTTCATTTTCTAAGAGCATCAATTTGCGTGA TTCTGGGTAGAAGATCG
p16	KIURA3-R4	GATATCACCGGTACGGAACCAGCCATCATCGGTAAAGGCATCGATAAGCTTGATATCG
p17	FAA4(dw)-F	CCTTTACCGATGATGGCTGGTTC
p18	FAA4(dw)-R	GATGTAACAAGACCGTTTTCTGGAG
**Primers for episomal plasmid construction for fatty acid production**		
p19	TPIp-F	GTTTAAAGATTACGGATATTTAACTTACTTAGAATAATG
p20	TPIp-R	CATTTTTAGTTTATGTATGTGTTTTTTGTAG
p21	PGK1p-F	CGCACAGATATTATAACATCTGCACAATAGG
p22	PGK1p-R	CATTTTGTTATATTTGTTGTAAAAAGTAGATAATTAC
p23	TEF1p-F	ATAGCTTCAAAATGTTTCTACTCCTTTTTTACTC
p24	TEF1p-R	CATTTTGTAATTAAAACTTAGATTAGATTGCTATGC
p25	TDH3p-F	CTCGAGTTTATCATTATCAATACTGCCATTTC
p26	TDH3p-R	GTTTGTTTATGTGTGTTTATTCGAAACTAAGTTCTTGGTG
p27	tHXT7p-F	GTATTCTTTGAAATGGCAGTATTGATAATGATAAACTCGAGCTCGTAGGAACAATTTCG
p28	tHXT7p-R	CATTTTTTGATTAAAATTAAAAAAACTTTTTGTTTTTGTG
p29	FBA1t-F	GTTAATTCAAATTAATTGATATAGTTTTTTAATGAG
p30	FBA1t-R	AGTAAGCTACTATGAAAGACTTTACAAAGAAC
p31	CYC1t-F	GATACCGTCGACCTCGAGTCATGTAATTAGTTATGTC

p32	CYC1t-R	GGGTACCGGCCGCAAATTAAAGCCTTCGAGCGTCC
p33	TDH2t-F	ATTTAACTCCTTAAGTTACTTTAATGATTTAGTTTTTA
p34	TDH2t-R	GCGAAAAGCCAATTAGTGTGATAC
p35	ADH1t-F	GCGAATTTCTTATGATTTATGATTTTTATTATTAAATAAG
p36	ADH1t-R	GCATATCTACAATTGGGTGAAATGGGGAGCGATTTG
p37	pYX212t-F	TAGGGCCCACAAGCTTACGCGTCGACCCGGGTATCC
p38	pYX212t-R	GCCGTAAACCACTAAATCGGAACCCTAAAGG
p39	RtACL-F1	CTATAACTACAAAAAACACATACATAAACTAAAAATGTCCGCAAAGCCTATCAGAG
p40	RtACL-R1	CTCATTAAAAAACTATATCAATTAATTTGAATTAACTTATTGTCTTTGTTGGACTAAAATT C
p41	RtME1-F1	CAAGAACTTAGTTTCGAATAAACACACATAAACAAACAAAATGCCTGCTCATTTTGCCC
p42	RtME1-R1	GACATAACTAATTACATGACTCGAGGTCGACGGTATCTCATACTTTTCTCAATGGTC
p43	LsME1-F1	CAAGAACTTAGTTTCGAATAAACACACATAAACAAACAAAATGGCCCCTAAATCCTCCA
p44	LSME1-R1	GTGACATAACTAATTACATGACTCGAGGTCGACGGTATCTCATACTTTTCTCAATGGTC
p45	MDH3t-F1	GTTTTTTTAATTTTAATCAAAAAATGGTCAAAGTCGCAATTCTTG
p46	MDH3t-R1	GGATACCCGGGTCGACGCGTAAGCTTGTGGGCCCTATCAAGAGTCTAGGATGAAACTC
p47	MDH3t-R2	TAAAAACTAAATCATTAAAGTAACTTAAGGAGTTAAATTCAAGAGTCTAGGATGAAAC
p48	CTP1-R	CTTATTTAATAATAAAAATCATAAATCATAAGAAATTCGCTCAGGCTAGCATAACTAAG
P49	CTP1-F	GAAGTAATTATCTACTTTTTACAACAAATATAACAAAATGTCCAGTAAAGCTACCAAAA G
P50	tTesA-F:	GCATAGCAATCTAATCTAAGTTTTAATTACAAAATGGCCGATACTTTGTTAATTTTG
P51	tTesA-R	GGATACCCGGGTCGACGCGTAAGCTTGTGGGCCCTATCAAGAATCGTGATTGACTAAT G
P52	PGK1p-R2	CTTATTTAATAATAAAAATCATAAATCATAAGAAATTCGCTTTGTTATATTTGTTGTAAA AAG
P53	fadM-F	GCATAGCAATCTAATCTAAGTTTTAATTACAAAATGCAAACCCAAATTAAGGTTAG
P54	fadM-R	GGATACCCGGGTCGACGCGTAAGCTTGTGGGCCCTATTATTTTACCATTTGTTCTAAC
**Primers for constructing pathways for alkane production**		
p55	CAR-F2	CTATAACTACAAAAAACACATACATAAACTAAAAATGTCACCTATCACCAGAGAAG
p56	CAR-R2	CTCATTAAAAAACTATATCAATTAATTTGAATTAACTCACAACAAACCCAACAATCTC
p57	npgA-F4	CACAAAAACAAAAAGTTTTTTTAATTTTAATCAAAAAATGGTGCAAGACACATCAAG
p58	npgA-R4	GGATACCCGGGTCGACGCGTAAGCTTGTGGGCCCTATTAGGATAGGCAATTACACACC
p59	ADH5up-F	GAAAAATGACTGATGTCTACAGGAC
p60	ADH5up-R	CATTATTCTAAGTAAGTTAAATATCCGTAATCTTTAAACCATGATGCTTTGATTTTGTAG ATATG
p61	ADH5dn-F	CGCCCTTGCTATGGGTTACAG
p62	ADH5dn-R	ACCTCTGGCGAAGAAATCTAAAGC
p63	TDH2t(tHXt 7p)-R	GAAGAACACGCAGGGGCCCGAAATTGTTCCTACGAGCGAAAAGCCAATTAGTGTGATA C
p64	npgA-R5	GCTTATATAAAAAGTAAAAATATATTCATCAAATTCGTTACAAAAGATTAGGATAGGCA ATTACACACC
p65	KIURA3-F6	CGAATTTGATGAATATATTTTTACTTTTTATATAAGCTATTTTGTAGATATTGACGTGATT CTGGGTAGAAGATCG

p66	KlURA3-R6	CGATACCAATGACCCTGTAACCCATAGCAAGGGCGCATCGATAAGCTTGATATCG
**Primers for constructing pathways for fatty alcohol production**		
p67	CAR-F1	CAAGAACTTAGTTTCGAATAAACACACATAAACAAACAAAATGTCACCTATCACCAGAGAAG
p68	CAR-R1	CTTATTTAATAATAAAAATCATAAATCATAAGAAATTCGCTTACAACAAACCCAACAATCTC
p69	npgA-F3	GCTTAAATCTATAACTACAAAAAACACATACATAAACTAAAAATGGTGCAAGACACATCAAG
p70	npgA-R3	CTCATTAAAAAACTATATCAATTAATTTGAATTAACTTAGGATAGGCAATTACACAC
p71	YjgB-F	GAAAGCATAGCAATCTAATCTAAGTTTTAATTACAAAATGTCAATGATAAAAAGTTAC
p72	YjgB-R	GGATACCCGGGTCGACGCGTAAGCTTGTGGGCCCTATTAGTGATGGTGATGGTGATGGTAATC
p73	ADH5-F	GAAAGCATAGCAATCTAATCTAAGTTTTAATTACAAAATGCCTTCGCAAGTCATTCCTGAAAAAC
p74	ADH5-R	GGATACCCGGGTCGACGCGTAAGCTTGTGGGCCCTATCATTTAGAAGTCTCAACAACATATC
p75	ADH6-F	GAAAGCATAGCAATCTAATCTAAGTTTTAATTACAAAATGTCTTATCCTGAGAAATTTGAAG
p76	ADH6-R	GGATACCCGGGTCGACGCGTAAGCTTGTGGGCCCTATTAGTCTGAAAATTCTTTGTCGTAGC
p77	ADH7-F	GAAAGCATAGCAATCTAATCTAAGTTTTAATTACAAAATGCTTTACCCAGAAAAATTTCAGG
p78	ADH7-R	GGATACCCGGGTCGACGCGTAAGCTTGTGGGCCCTATTATTTATGGAATTTCTTATCATAATC
p79	SFA1-F	GAAAGCATAGCAATCTAATCTAAGTTTTAATTACAAAATGTCCGCCGCTACTGTTGGTAAAC
p80	SFA1-R	GGATACCCGGGTCGACGCGTAAGCTTGTGGGCCCTACTATTTTATTTCATCAGACTTCAAGACG
p81	CAR-R3:	CCACCCAACAAACCCAACAATCTCAAATC
p82	ADH5-F2	GTATCAGATTTGAGATTGTTGGGTTTGTTGGGTGGTGGTTCTGGTGGTGGTTCTATGCCTTCGCAAGTCATTCCTG
p83	ADH5-R2	CTTATTTAATAATAAAAATCATAAATCATAAGAAATTCGCTTATTTAGAAGTCTCAACAACATATC
p84	ADH5-F3	CAAGAACTTAGTTTCGAATAAACACACATAAACAAACAAAATGCCTTCGCAAGTCATTCCTG
p85	ADH5-R3	CTAATCTTTCTTCTCTGGTGATAGGTGACATAGAACCACCACCAGAACCACCACCTTTAGAAGTCTCAACAACATATC
p86	CAR-F3	GTGGTTCTATGTCACCTATCACCAGAGAAG
p87	ADH5-F4	CACAAAAACAAAAAGTTTTTTTAATTTTAATCAAAAAATGCCTTCGCAAGTCATTCCTGAAAAAC
p88	ADH5-R4	GACATAACTAATTACATGACTCGAGGTCGACGGTATCTCATTTAGAAGTCTCAACAACATATC
p89	FaCoAR-F1	GCATAGCAATCTAATCTAAGTTTTAATTACAAAATGAATTATTTCTTGACAGGTG
p90	FaCoAR-R1	GGATACCCGGGTCGACGCGTAAGCTTGTGGGCCCTATTACCAATAGATACCTCTCA
p91	FaCoAR -F2	GTATCAGATTTGAGATTGTTGGGTTTGTTGGGTGGTGGTTCTGGTGGTGGTTCTATGAATTATTTCTTGACAGG
p92	FaCoAR -R2	CTTATTTAATAATAAAAATCATAAATCATAAGAAATTCGCTTACCAATAGATACCTCTCATAATG

P93	Gal10t-F2	CTGGGCTGCAGGAATTCGATATCAAGCTTATCGATGGGAGACACTATTGAGGGTACGGAG
P94	Gal10t-R	GTTTCACCGTTTTTCAAGGTTACAC
P95	MmCAR-F6	CATGATAAAAAAAAACAGTTGAATATTCCCTCAAAAATGTCACCTATCACCAGAGAAGAAAG
P96	npgA-F6	GAGAAAATAAAAGTAAAAAGGTAGGGCAACACATAGTATGGTGCAAGACACATCAAG
P97	Gal7p-F	TTTGCCAGCTTACTATCCTTCTTG
P98	Gal7p-R	CATTTTTGAGGGAATATTCAACTG
P99	Gal3p-F	GTGCATATTTTCAAGAAGGATAGTAAGCTGGCAAATTGCTAGCCTTTTCTCGGTCTTGC
P100	Gal3p-R	ACTATGTGTTGCCCTACCTTTTTAC
P101	FBA1t-URA3-R1	CATTCATATCATATTTTTTCTATTAACTGCCTGGTTTCTTTTAAATTTTTATTGGTTGTCGCATCGATAAGCTTGATATCG
P102	URA3(Gal7)-F	CGAGGTCCTCCTTCACCATTTGGTTAAATTGGCTGTGATTCTGGGTAGAAGATCG
P103	Gal7(dn)-F	AGCCAATTTAACCAAATGGTGAAG
P104	Gal7(dn)-R	CAGTCTTTGTAGATAATGAATCTG
**Primers for genomic integration for free fatty acid production**		
P105	His3(up)-F	CTCTTGGCCTCCTCTAGTACACTC
P106	His-R3	GCAGAAAAGACTAATAATTCTTAGTTAAAAGCACTCTACATAAGAACACCTTTGGTGG
P107	ENO2t-F	AGTGCTTTTAACTAAGAATTATTAGTC
P108	ENO2t-R	AGGTATCATCTCCATCTCCCATATGCATATCA
P109	ENO2t-TPIp-F	CCACAGTGATATGCATATGGGAGATGGAGATGATACCTGATCTACGTATGGTCATTCTTC
P110	’TesA-R2	CGTATGCTGCAGCTTTAAATAATCGGTGTCATCAAGAATCGTGATTGACTAATG
P111	His3t-F	GACACCGATTATTTAAAGCTGCAG
P112	His3t-R	CTGTTATTTCTGGCACTTCTTGG
P114	MmACL-F	CTATAACTACAAAAAACACATACATAAACTAAAAATGTCCGCTAAAGCTATTTCC
P115	MmACL-R	GGATACCCGGGTCGACGCGTAAGCTTGTGGGCCCTATTACATACTCATGTGTTCAGG
P116	HsACL-F	CTATAACTACAAAAAACACATACATAAACTAAAAATGTCCGCAAAAGCCATTTCC
P117	HsACL-R	GGATACCCGGGTCGACGCGTAAGCTTGTGGGCCCTATTACATACTCATGTGTTCAGG
P118	URA3(up)-F	AAACGACGTTGAAATTGAGGCTACTGCG
P119	URA3(up)-R	GAAGAAGAATGACCATACGTAGATCCCCAATTCGGACTAGGATGAGTAGCAGCACGTTCC
P120	RtFAS1-F	CTATAACTACAAAAAACACATACATAAACTAAAAATGAACGGCCGAGCGACGCGGAG
P121	RtFAS1-R	CTCATTAAAAAACTATATCAATTAATTTGAATTAACTCAGAGCCCGCCGAAGACGTCGAG
P122	RtFAS2-F	GAAAGCATAGCAATCTAATCTAAGTTTTAATTACAAAATGGTCGCGGCGCAGGACTTGC
P123	RtFAS2-R	GACATAACTAATTACATGACTCGAGGTCGACGGTATCCTACTTCTGGGCGATGACGACG
P124	TEF1p(URA3)-F:	GTTTTGCTGGCCGCATCTTCTCAAATATGCTTCCCAGCCATAGCTTCAAAATGTTTCTACTCC
P125	Amdsym-F	GAGTAAAAAAGGAGTAGAAACATTTTGAAGCTATAAGCTTCGTACGCTGCAGGTCG
P126	Amdsym-R	CTGGCCGCATCTTCTCAAATATGCTTCCCCGACTCACTATAGGGAGACCG
P127	URA3(dn)-F	GGGAAGCATATTTGAGAAGATGCGGC

P128	URA3(dn)-R	GGAAACGCTGCCCTACACGTTCGC
P129	ACC1(up)-F	CGTTACGCCCTCCAGAGTCACC
P130	ACC1(up)-R	CTGGGCTGCAGGAATTCGATATCAAGCTTATCGATGCTAGGCTATACTGTGCCAGAATACG
P131	TEF1p(ACC1)-F	CATCCAATGCAGACCGATCTTCTACCCAGAATCACATAGCTTCAAAATGTTTCTACTCC
P132	TEF1p(ACC1)-R	CTGTGGAGAAGACTCGAATAAGCTTTCTTCGCTCATTTTGTAATTAAAACTTAGATTAG
P133	ACC1-F	ATGAGCGAAGAAAGCTTATTCG
P134	ACC1(In)-R	GTACCACCTGGCACTTCAATG

Supplementary Table 5. Codon optimized genes used in this study.

Synthesized genes	Sequence (5'-3')
RtACL	ATGTCCGCAAAGCCTATCAGAGAATACGACGCCAAATTGTTGTTAGCCTATCACTTAGCAAG<br>AGCCCCTACCGCAGGTTCCAAAGCAGTTGCAAGAGATGGTTTTCAATCTCCAGAAGTAAAA<br>GTTGCCCAAGTCTCATGGGACCCTGAAACCAATCAAGTAACTCCAGATGCTGCATTGCCTCA<br>TTGGGTTTTCACTGAAAAATTGGTTGTCAAGCCAGATCAATTGATTAAAAGACGTGGTAAAG<br>CAGGTTTGTTAGCCTTAAACAAAACTTGGGCTGAAGGTAAACAATGGATAGCCGAAAGAGC<br>TGGTAAACAAGTCCAAGTAGAAAAGACTACAGGTACATTGAACAACTTCATCGTTGAACCAT<br>TCTGTCCACATCCTTCCGATGCTGAATACTACATTTGCATCAACAGTGTCAGAGAAGGTGAC<br>GTAATTTTGTTTACTCACGAAGGTGGTGTTGATGTCGGTGACGTTGACGCCAAAGCATTGAC<br>TTTGTTAGTACCAGTTGGTGGTGAATTGCCTTCCAGAGATGAAATCAGAAGTCAATTGTTGA<br>AGCATGTTACAGGTGCAGAAAGACAAGAAGCCTTAATAGACTACATCATCAGATTGTACTCC<br>GTCTACGTAGATTTGCACTTTGCTTACTTAGAAATCAATCCATTGGTTGCAGTCGAAAACCCT<br>TCTACTGGTAAAACAGATATTTTCTATTTGGATATGGCCGCTAAGTTGGACCAAACTGCTGA<br>ATACGTAGTTGGTCCAAAATGGGCAATAGCCAGAGATCCTTCAATCATTAATCCAGCAGCCG<br>CTCCTATGTCTAACGGTAAAATCTCAGCTGATAAGGGTCCACCTATGTTTTGGCCACCTCCAT<br>TCGGTAGAGACTTAACTAAGGAAGAAGCATATATTGCCAAGTTGGATGGTTCTACAGGTGC<br>CTCATTGAAATTGACCGTATTAAATGCTGAAGGTAGAATATGGACAATGGTTGCTGGTGGT<br>GGTGCATCCGTCGTATATAGTGATGCTATCGCAGCTCATGGTTTTGCCCACGAATTGGCTAA<br>TTATGGTGAATACTCTGGTGCACCAACTCAAACACAAACCTATGAATACGCCAAAACTATTTT<br>GGATTTGATGACCAGAGGTACTCCAAACCCTCAGGGTAAATTGTTGTTTATTGGTGGTGGTA<br>TTGCAAATTTTACTAACGTTGCTGCAACATTCAAAGGTATCATCACAGCTTTGAAGGAATACC<br>AACATAGATTGCAAGAACACAAAGTTAGAATCTTCGTCAGAAGAGGTGGTCCAAATTACCA<br>AGAAGGTTTAAAGGCTATGAGATTGTTAGGTGAAACTTTGGGTGTAGAAATCCAAGTTTTC<br>GGTCCAGAAACACATATTACCTCTATAGTTCCTTTGGGTTTAGGTTTGATTAAATCAGTTGAT<br>GACGCCTTAAAGGTCCCAGGTGCTAGAGCTGCTGCTGATGCAACTGGTACATTAACCCCAGT<br>TCCTGGTTCCCCAAAAAGTAGAGCCGCTCAATTGCCTACAGGTGCATCTACCCCATCAAGAC<br>AACAACCTCAAGATAACATAGTATCCTTTAGTGATAAAGTTCATGCTCCAGACTCTGGTAGA<br>CCTTGGTATAGACCTTTCGATGAAACCACTAGAAGTATAGTTTACGGTTTACAACCTAGAGC<br>TATCCAAGGCATGTTGGATTTTGACTTCGCATGTGGTAGAGAAACACCATCTGTCGCAGCTA<br>TGGTTTATCCTTTTGGTGGTCATCACGTTCAAAAATTCTACTGGGGTACTAAGGAAACATTGT<br>TGCCAGTTTTTACTTCAATGAAGGAAGCTGTCGCAAAGTGCCCTGATGCCGACGTTGTCGTA<br>AACTTCGCTTCTTCAAGATCAGTTTACCAATCTACTTTGGAAGCATTGGAATTTCCACAAATC<br>AAAGCCATTGCTTTGATAGCTGAAGGTGTTCCTGAAAGACATGCAAGAGAAATTTTACACTT<br>GGCCAAAAAGAAAGAAGTAATTATCATCGGTCCAGCTACTGTTGGTGGTATTAAACCAGGT<br>TGTTTCAGAATCGGTAACACAGGTGGTATGAACGAAAACATCTTGTCCAGTAAATTGTATAG<br>AGCTGGTTCTGTAGGTTACGTTTCTAAGTCAGGTGGCATGTCTAACGAATTAAACAACATAT<br>TATCATTGACAACCGACGGTGCTTATGAAGGTATCGCAATTGGTGGTGACAGATACCCAGG<br>TACTACTTTTATTGACCATTTGTTGAGATACGAAGCAGATCCTAACTGTAAGATGTTGGTTTT<br>GTTGGGTGAAGTCGGTGGTGTAGAAGAATACAGAGTTATTGAAGCTGTCAAATCTGGTCAA<br>ATTAAGAAACCAATCGTCGCATGGGCCATAGGTACTTGCGCAAAGATGTTTGCCACAGACG<br>TACAATTCGGTCACGCTGGTTCTATGGCTAATTCTGATTTGGAAACAGCTGAAGCTAAAAAT<br>AACGCAATGAGAGCTGCTGGTTTTATTGTTCCTCCAACCTTCGAAGAATTGCCACAAGTTTTG<br>GCTGAAACATACCAAAAATTGGTCGGTGACGGTACTATTCAACCAAAGCCTGAAGTTCCTCC<br>ACCTCAAATACCAATGGATTACAATTGGGCACAAACATTGGGTATGGTCAGAAAACCTGCC<br>GCTTTTATCTCCACCATTAGTGACGAAAGAGGTCAAGAATTGTTATATGCTGGTATGCCAAT<br>TTCTAAGGTTTTCGAAGAAGATATAGGTATCGGTGGTGTTGTCTCATTGTTGTGGTTCAAGA

	GAAGATTACCAGCCTACGCTACTAAGTTCTTGGAAATGGTTTTGATGTTGACTGCTGATCAT<br>GGTCCTGCCGTCTCTGGTGCTATGACCACTGTAATTACAACCAGAGCCGGTAAAGATTTGGT<br>ATCTTCATTAGTTGCTGGTTTGTTAACTATAGGTGACAGATTTGGTGGTGCTTTAGACGGTG<br>CAGCCCAAGAGTTTACTAGAGCTTTCGAAGCAGGTTTGACTCCAAGAGAATTTGTTGATTCT<br>ATGAGAAAGGCAAATAAGTTAATACCAGGTATCGGTCATAAGGTCAAATCAAAGGCTAATC<br>CTGATAAAAGAGTTGAATTGGTCAAAAACTACGTTTTTAAACACTTCCCATCCGCAAAGTTG<br>TTAGAATACGCATTAGCCGTAGAAGATGTTACCAGTGCTAAGAAAGACACTTTGATCTTGAA<br>CGTTGATGGTGCTATTGCAGTCTCCTTTGTAGATTTGTTGAAAAATTCTGGTGCCTTCACCGC<br>TGAAGAAGCTGCTGAATACATGAAGATCGGTACTTTGAACGGTTTGTTCGTTTTGGGTAGAT<br>CAATCGGTTTCATAGCACATCACTTGGATCAAAAGAGATTGAAGCAACCATTATACAGACAT<br>CCTGCTGACGACATTTTCATCCAACCATTCAACACTGACAGAATTTTAGTCCAACAAAGACAA<br>TAA
MmACL	ATGTCCGCTAAAGCTATTTCCGAACAAACTGGTAAAGAATTATTATACAAGTACATTTGCAC<br>CACCTCAGCCATACAAAACAGATTCAAGTATGCAAGAGTTACACCAGATACCGACTGGGCCC<br>ATTTGTTACAAGATCACCCTTGGTTGTTATCTCAATCATTGGTTGTCAAACCTGACCAATTGA<br>TTAAAAGACGTGGTAAATTGGGTTTAGTCGGTGTAAACTTGAGTTTAGATGGTGTTAAGTCT<br>TGGTTGAAGCCAAGATTAGGTCATGAAGCTACAGTTGGTAAAGCAAAGGGTTTCTTGAAAA<br>ATTTCTTGATCGAACCATTCGTACCTCACTCACAAGCTGAAGAATTTTACGTTTGTATCTATG<br>CAACTAGAGAAGGTGACTATGTCTTGTTTCATCACGAAGGTGGTGTTGACGTCGGTGACGTT<br>GACGCCAAAGCTCAAAAGTTGTTAGTAGGTGTTGATGAAAAGTTAAACACAGAAGACATCA<br>AGAGACATTTGTTGGTACACGCCCCAGAAGATAAAAAGGAAGTTTTGGCTTCCTTTATAAGT<br>GGTTTGTTTAATTTCTACGAAGATTTGTACTTCACCTACTTGGAAATTAACCCTTTAGTAGTTA<br>CTAAGGATGGTGTCTATATATTGGACTTAGCTGCAAAAGTAGATGCAACTGCCGACTACATC<br>TGTAAGGTTAAGTGGGGTGACATTGAATTTCCACCTCCATTCGGTAGAGAAGCATATCCAGA<br>AGAAGCCTACATTGCTGATTTGGACGCAAAATCTGGTGCCTCATTGAAGTTAACATTGTTGA<br>ACCCTAAGGGTAGAATATGGACTATGGTTGCTGGTGGTGGTGCAAGTGTCGTATATTCTGA<br>TACAATCTGCGACTTGGGTGGTGTTAACGAATTAGCTAACTACGGTGAATACTCAGGTGCAC<br>CATCCGAACAACAAACTTATGATTACGCTAAGACCATCTTGAGTTTAATGACTAGAGAAAAG<br>CATCCTGAAGGTAAAATTTTGATCATCGGTGGTTCTATAGCAAACTTCACTAACGTTGCCGCT<br>ACATTCAAGGGTATAGTCAGAGCTATCAGAGATTATCAAGGTCCATTGAAGGAACACGAAG<br>TTACAATATTCGTCAGAAGAGGTGGTCCTAACTACCAAGAAGGTTTAAGAGTAATGGGTGA<br>AGTTGGTAAAACTACAGGTATCCCAATTCATGTATTTGGTACTGAAACACACATGACTGCCA<br>TCGTTGGTATGGCTTTAGGTCATAGACCAATTCCTAATCAACCTCCAACAGCAGCCCACACC<br>GCCAATTTCTTGTTAAACGCTTCCGGTAGTACCTCTACTCCAGCACCATCAAGAACTGCCTCA<br>TTCTCCGAAAGTAGAGCTGATGAAGTTGCTCCAGCTAAGAAAGCAAAACCAGCCATGCCTC<br>AAGACTCCGTTCCAAGTCCTAGATCATTGCAAGGTAAATCAGCAACATTATTTTCCAGACAT<br>ACCAAAGCCATTGTATGGGGTATGCAAACAAGAGCTGTTCAAGGCATGTTGGATTTCGACT<br>ATGTTTGTAGTAGAGATGAACCATCTGTCGCTGCAATGGTATATCCTTTTACCGGTGACCAT<br>AAACAAAAGTTCTACTGGGGTCACAAGGAAATATTAATCCCAGTTTTTAAAAACATGGCCGA<br>TGCTATGAAAAAGCATCCTGAAGTTGATGTATTGATTAACTTCGCTTCATTAAGATCCGCTTA<br>TGATTCTACTATGGAAACAATGAACTACGCACAAATTAGAACCATAGCTATCATTGCAGAAG<br>GTATACCAGAAGCATTGACTAGAAAGTTAATCAAAAAGGCCGATCAAAAAGGTGTCACTAT<br>AATCGGTCCAGCTACAGTAGGTGGTATAAAACCTGGTTGTTTTAAGATCGGTAATACTGGTG<br>GCATGTTGGATAACATATTGGCATCAAAATTGTATAGACCAGGTTCCGTAGCTTACGTTTCA<br>AGAAGCGGTGGTATGAGTAACGAATTGAACAACATAATTTCAAGAACCACTGATGGTGTTT<br>ATGAAGGTGTCGCTATTGGTGGTGACAGATACCCAGGTTCTACTTTTATGGATCATGTTTTG<br>AGATATCAAGACACACCTGGTGTCAAAATGATCGTTGTCTTAGGTGAAATAGGTGGTACTG<br>AAGAATACAAAATTTGCAGAGGTATAAAGGAAGGTAGATTGACAAAACCAGTAGTTTGTTG

	GTGCATTGGTACTTGTGCAACTATGTTTTCTTCAGAAGTTCAATTCGGTCATGCAGGTGCCTG<br>CGCTAATCAAGCATCTGAAACAGCAGTTGCCAAAAACCAAGCCTTAAAGGAAGCTGGTGTT<br>TTTGTCCCTAGATCATTCGATGAATTGGGTGAAATCATTCAATCCGTATATGAAGACTTAGTT<br>GCCAAGGGTGCTATTGTCCCAGCTCAAGAAGTACCTCCACCTACTGTTCCTATGGATTACTCA<br>TGGGCAAGAGAATTGGGTTTGATCAGAAAGCCAGCTAGTTTTATGACCTCTATCTGTGATGA<br>AAGAGGTCAAGAATTGATCTATGCTGGTATGCCTATCACTGAAGTCTTCAAGGAAGAAATG<br>GGTATCGGTGGTGTATTGGGTTTGTTGTGGTTCCAAAGAAGATTACCAAAGTACTCATGTCA<br>ATTCATAGAAATGTGCTTAATGGTTACAGCTGATCATGGTCCAGCTGTTTCTGGTGCCCACA<br>ACACCATAATCTGCGCTAGAGCAGGTAAAGATTTGGTTTCTTCTTTGACCTCTGGTTTGTTAA<br>CTATTGGTGACAGATTTGGTGGTGCATTGGACGCCGCTGCAAAAATGTTTTCAAAGGCTTTC<br>GATTCCGGTATAATCCCAATGGAATTTGTTAATAAGATGAAAAAGGAGGGTAAATTAATCAT<br>GGGTATCGGTCATCGTGTTAAGTCAATTAATAACCCTGATATGAGAGTCCAAATATTGAAGG<br>ACTTCGTAAAGCAACACTTCCCAGCAACACCTTTGTTAGATTACGCCTTAGAAGTTGAAAAG<br>ATTACAACCTCTAAAAAGCCAAATTTGATCTTGAACGTTGATGGTTTTATAGGTGTCGCTTTC<br>GTAGACATGTTAAGAAACTGTGGTTCTTTTACTAGAGAAGAAGCCGATGAATATGTTGACAT<br>TGGTGCTTTGAATGGTATATTTGTCTTAGGTAGATCAATGGGTTTTATTGGTCATTACTTGGA<br>TCAAAAGAGATTAAAGCAAGGTTTGTATAGACACCCTTGGGACGATATTTCCTACGTTTTGC<br>CTGAACACATGAGTATGTAA
HsACL	ATGTCCGCAAAAGCCATTTCCGAACAAACTGGTAAAGAATTATTATACAAGTTCATCTGCAC<br>AACCTCAGCCATACAAAACAGATTCAAGTATGCAAGAGTTACTCCAGATACAGACTGGGCC<br>AGATTGTTACAAGATCATCCTTGGTTGTTATCACAAAACTTGGTTGTCAAGCCTGACCAATTG<br>ATTAAAAGACGTGGTAAATTGGGTTTAGTAGGTGTTAATTTGACATTAGATGGTGTTAAGTC<br>CTGGTTGAAGCCAAGATTAGGTCAAGAAGCAACCGTCGGTAAAGCCACTGGTTTCTTGAAA<br>AATTTCTTGATCGAACCATTCGTACCTCATTCTCAAGCTGAAGAATTTTACGTTTGTATATAC<br>GCAACTAGAGAAGGTGACTATGTCTTGTTTCATCACGAAGGTGGTGTTGATGTAGGTGACG<br>TAGACGCCAAAGCTCAAAAGTTGTTAGTTGGTGTCGATGAAAAATTGAACCCAGAAGACAT<br>TAAAAAGCATTTGTTGGTTCACGCCCCTGAAGATAAAAAGGAAATATTGGCTTCTTTTATCTC<br>AGGTTTGTTTAATTTCTACGAAGATTTGTACTTCACTTACTTGGAAATTAACCCATTGGTAGT<br>TACAAAGGATGGTGTATACGTTTTGGACTTAGCTGCAAAGGTCGATGCAACAGCCGACTAC<br>ATTTGTAAAGTAAAGTGGGGTGACATAGAATTTCCACCTCCATTCGGTAGAGAAGCATATCC<br>AGAAGAAGCCTACATTGCTGATTTGGACGCCAAATCCGGTGCTAGTTTGAAGTTAACCTTGT<br>TGAACCCTAAAGGTAGAATCTGGACTATGGTTGCAGGTGGTGGTGCCTCAGTCGTATATTCC<br>GATACTATTTGCGACTTGGGTGGTGTTAACGAATTAGCTAACTACGGTGAATACAGTGGTGC<br>ACCATCTGAACAACAAACCTATGATTACGCTAAGACTATCTTGAGTTTAATGACAAGAGAAA<br>AGCATCCTGATGGTAAAATTTTGATCATCGGTGGTTCTATCGCTAACTTCACAAACGTCGCC<br>GCTACCTTCAAAGGTATAGTAAGAGCAATCAGAGATTACCAAGGTCCATTGAAGGAACACG<br>AAGTAACCATTTTTGTTAGAAGAGGTGGTCCTAACTACCAAGAAGGTTTAAGAGTCATGGG<br>TGAAGTAGGTAAAACTACAGGTATCCCAATTCATGTATTTGGTACTGAAACTCACATGACTG<br>CTATTGTTGGTATGGCATTAGGTCATAGACCAATACCTAATCAACCTCCAACTGCTGCTCACA<br>CAGCTAATTTCTTGTTAAACGCATCTGGTTCAACATCCACCCCAGCCCCATCAAGAACAGCTA<br>GTTTCTCTGAATCAAGAGCTGATGAAGTTGCTCCAGCTAAGAAAGCAAAACCAGCCATGCCT<br>CAAGACTCTGTTCCATCACCTAGATCCTTGCAAGGTAAAAGTACCACTTTGTTTTCAAGACAT<br>ACAAAGGCAATTGTTTGGGGTATGCAAACCAGAGCCGTCCAAGGCATGTTGGATTTCGACT<br>ATGTTTGTTCAAGAGATGAACCATCCGTTGCTGCAATGGTCTATCCTTTTACTGGTGACCATA<br>AACAAAAGTTCTACTGGGGTCACAAGGAAATATTAATCCCAGTTTTTAAAAACATGGCCGAT<br>GCTATGAGAAAGCATCCTGAAGTTGATGTATTGATTAACTTCGCAAGTTTAAGATCAGCCTA<br>TGATTCAACTATGGAAACTATGAACTACGCTCAAATCAGAACTATTGCTATCATTGCAGAAG<br>GTATCCCAGAAGCATTGACAAGAAAATTAATTAAAAAGGCAGATCAAAAGGGTGTAACCAT<br>AATCGGTCCAGCAACTGTTGGTGGTATCAAACCTGGTTGTTTTAAGATTGGTAATACAGGTG

	GCATGTTGGATAACATATTGGCTTCAAAATTGTATAGACCAGGTTCCGTCGCATACGTATCC<br>AGAAGTGGTGGTATGAGTAACGAATTAAACAACATAATTTCAAGAACAACCGATGGTGTAT<br>ATGAAGGTGTTGCTATTGGTGGTGACAGATACCCAGGTTCTACTTTTATGGATCATGTTTTG<br>AGATATCAAGACACCCCTGGTGTCAAAATGATCGTTGTCTTAGGTGAAATAGGTGGTACAG<br>AAGAATACAAAATTTGTAGAGGTATTAAGGAAGGTAGATTGACCAAACCAATTGTTTGTTG<br>GTGCATAGGTACATGTGCTACCATGTTTTCTTCAGAAGTTCAATTCGGTCACGCAGGTGCCT<br>GCGCTAATCAAGCATCTGAAACAGCAGTTGCCAAAAACCAAGCATTGAAGGAAGCAGGTGT<br>TTTTGTCCCTAGATCATTCGATGAATTGGGTGAAATCATTCAATCCGTCTATGAAGACTTAGT<br>AGCCAATGGTGTAATTGTTCCAGCTCAAGAAGTTCCTCCACCTACTGTCCCTATGGATTACTC<br>TTGGGCTAGAGAATTGGGTTTAATCAGAAAACCAGCTTCTTTTATGACTTCCATTTGTGATGA<br>AAGAGGTCAAGAATTGATCTATGCTGGTATGCCTATCACAGAAGTTTTCAAGGAAGAAATG<br>GGTATAGGTGGTGTCTTGGGTTTGTTGTGGTTCCAAAAGAGATTGCCAAAGTACTCATGTCA<br>ATTCATTGAAATGTGCTTAATGGTCACCGCTGATCATGGTCCTGCCGTATCCGGTGCTCACA<br>ACACTATAATCTGCGCTAGAGCAGGTAAAGATTTGGTTTCTTCTTTGACTTCAGGTTTGTTAA<br>CAATTGGTGACAGATTTGGTGGTGCTTTGGACGCCGCTGCAAAGATGTTTAGTAAGGCATTC<br>GATTCTGGTATAATCCCAATGGAATTTGTTAATAAGATGAAAAAGGAGGGTAAATTAATCAT<br>GGGTATCGGTCATCGTGTTAAGTCTATAAATAACCCTGATATGAGAGTACAAATCTTGAAGG<br>ACTATGTTAGACAACACTTTCCAGCAACACCTTTGTTAGATTACGCCTTAGAAGTTGAAAAG<br>ATTACTACATCTAAGAAACCAAATTTGATCTTGAACGTTGATGGTTTGATCGGTGTTGCTTTT<br>GTTGATATGTTAAGAAACTGTGGTAGTTTCACTAGAGAAGAAGCCGATGAATATATTGACAT<br>CGGTGCTTTGAACGGTATCTTCGTTTTGGGTAGATCAATGGGTTTTATTGGTCATTACTTGGA<br>TCAAAAGAGATTAAAGCAAGGTTTGTATAGACACCCTTGGGATGATATTTCCTACGTTTTGC<br>CTGAACACATGAGTATGTAA
RtME	ATGCCTGCTCATTTTGCCCCTTCACAACCATTACAAGGTGGTCCATCCCCTTCACAATTGGGT<br>CCTAAAGAATTATTGATAGAAAGAGCATTGACAAGATTGAGATCAATCCCAAACGATTTGG<br>AAAAATATACCTTTTTGGCCGGTTTAAGAGGTAGAAATCCTGATGTCTTCTACGGTTTAGTA<br>GGTGGTAACATGAAGGAATGTTGCCCAATTATCTATACTCCTGTTATAGGTTTAGCTTGTCA<br>AAATTGGTCCTTGATCCATCCACCTCCACCTGAAAGTGATCCAACAATTGACGCATTGTATTT<br>GTCTTACTCAGATTTGCCAAACTTACCTCAATTGATCGGTGGTTTGAAGACTAGATTGCCTCA<br>CGATCAAATGCAAATCTCCGTTGTCACAGACGGTAGTAGAGTATTGGGTTTGGGTGACTTG<br>GGTGTTGGTGGTATGGGTATATCTCAGGGTAAATTGTCATTATACGTTGCTGCTGGTGGTGT<br>CAATCCAAAGGCCACTTTACCTATCGCTATTGATTTTGGTACTGACAACGAAACTTTGTTAGC<br>TGATCCATTGTACGTTGGTCAAAGAATTAGAAGATTATCTCAAGAAAAGTGTTTGGAGTTTA<br>TGGAAGTTTTCATGAGATGCATGCATGAAACCTTCCCAAATATGGTTATTCAACACGAAGAC<br>TGGCAAACTCCATTGGCTTTCCCTTTGTTGCATAAGAACAGAGATTTGTACCCTTGTTTCAAC<br>GATGACATTCAAGGTACTGGTGCAGTAGTTTTAGCAGGTGCCATAAGAGCTTTTCACTTAAA<br>CGGTGTTGCATTGAAGGATCAAAAGATTTTGTTTTTCGGTGCCGGTTCTTCAGGTGTTGGTG<br>TCGCTGAAACAATATGCAAGTACTTCGAATTGCAAGGCATGTCTGAAGACGAAGCCAAATC<br>AAAGTTCTGGTTGGTAGATTCAAAGGGTTTGGTTGCTCATAATAGAGGTGACACATTACCAT<br>CTCACAAAAAGTATTTGGCAAGATCAGAACCAGATGCCCCTAAATTGAGAACCTTGAAGGA<br>AGTCGTAGAACATGTTCAACCAACTGCTTTGTTAGGTTTATCTACAGTCGGTGGTACTTTTAC<br>AAAGGAAATCTTGGAAGCTATGGCAACTTACAATAAGAGACCAATTGTCTTTGCTTTATCAA<br>ACCCTGTAGCCCAAGCTGAATGTACCTTCGAAGAAGCTGTTGAAGGTACTGACGGTAGAGT<br>CTTGTACGCATCCGGTAGTCCATTCGATCCTGTTGAATACAAGGGTAAAAGATACGAACCAG<br>GTCAAGGTAATAACATGTATATCTTCCCTGGTTTAGGTATTGGTGCTATATTGGCAAGAGTC<br>TCCAAAATTCCAGAAGAATTAGTACATGCATCCGCCCAAGGTTTAGCAGACAGTTTGACACC<br>AGAAGAAACCGCCAGACACTTGTTGTACCCTGATATCGAAAGAATTAGAGAAGTTTCTATAA<br>AAATCGCTGTAACAGTTATACAAGCCGCTCAAAAGTTAGGTGTTGATAGAAACGAAGAATT<br>GCGTGGTAAATCCAGTGCAGAAATTGAAGCCTATGTCAGAAAAGGTATGTATCACCCATTAT

	TAGAAGCAGAACAACAAGCACAATGA
LsME	ATGGCCCCTAAATCCTCCACCAGAGTTCCATTATCCGTCAAAGGTCCAATAGACTGC<br>CCTTATGAAGGTAAAGAAATGTTAAACTTACCTCAATTCAATAGAGGTACAGCCTTC<br>ACCGCTGAAGAAAGAGATTTGTTTAATTTGGTCGGTAATTTGCCAGCTGCATTACAA<br>ACTTTGCAAAATCAAGTAGACAGAGCCTATGATCAATACTCTTCAATTTCTACAGCTT<br>TGGGTAAAAACACCTTTTTAATGTCATTGAAGGTCCAAAACGAAGTATTGTACTTCA<br>AATTGTTACAAGATCATTTGAAGGAAATGTTCTCAATCATCTATACTCCAACAGAATC<br>TGAAGCTATCGAACATTATTCAAGATTGTTTAGAAGACCAGAAGGTTGTTTCTTGAA<br>CATCAACCACCCAGAATACATCGAAAGATCCTTAGCCGCTTGGGGTACAGAAGAAG<br>ATATTGACTACATCATTGTTAGTGACGGTGAAGAAATTTTGGGTATAGGTGACCAA<br>GGTGTCGGTGCTATAGGTATCTCCAGTGCAAAAGCCGTATTAATGACCTTGTGTGCA<br>GGTGTTCATCCATCAAGATGCATTCCAGTTGCCTTAGACGTCGGTACTGATAACGAA<br>CAATTGTTAGAAGATGAATTATATTTGGGTAACAGACACAACAGAGTTAGAGGTGG<br>TAGATACGATAAATTTGTCGATGACTTCGTTCAATGTGTCAAAAAGTTATATCCAAG<br>AGCCGTTTTGCATTTTGAAGACTTCGGTTTACCTAACGCAAGAAGATTGTTAGATAC<br>TTACAGACCAAGATTGGCTTGCTTTAATGATGACGTCCAAGGTACTGGTGCAGTAAC<br>TTTAGCAGCCTTGTCTTCAGCTGTCAGAGTAGCAGGTATCGATTTTAGAGACTTGAG<br>AACAGTTATTTTCGGTGCAGGTACTGCCGGTACAGGTATAGCTGACCAATTAAGAG<br>ATTTCTTGAATACCCAAGGTATCTCTAAACAACAAGTTATCGACCATATTTGGTTGGT<br>CGATAAGCCTGGTTTGTTATTGAAATCCATGCACGATAAGTTGACTAGTGCACAAAG<br>ACCATACGCTGCATCTGATGACAGATGGAAGGAAATAGATACAAAGTCCTTAAGTG<br>AAATCGTTAAGAAAGTTAAGCCTCACGTTTTGATTGGTTGTTCTACTAAACCAAAGG<br>CCTTCAACGAAGCTGTTTTAAGAGAAATGGCTAAGCATGTAGAAAGACCAATCGTTT<br>TCCCTTTGTCAAACCCAACTAGATTGCACGAAGCTACACCAGCTGAAATTTTTAAATA<br>CACCGATGGTAAAGCATTGGTAGCTACTGGTTCCCCATTCGATCCTGTTGACGGTAA<br>AGAAATCGCTGAAAACAACAACTGCTTCGTTTACCCAGGTATCGGTATGGGTTCTAT<br>TTTGTCAAGAGCAGATAGAGTTACCGAAACTATGATAGCCGCTGTTGTCAAAGAATT<br>AGCATCCTTGGCCCCTAGTGAAAAAGATCCAACAGGTGCATTATTGCCTGATGTTGC<br>CGACATAAGAGATATCTCTGCTAAAATTGCTACAGCAGTAGTTTTGCAAGCATTGGA<br>AGAAGGTACTGCAAGAGTCGAAGAAATAGAAGGTATTAAAGTTCCAAGAGATAGA<br>GACCATTGTTTGGAATGGGTAAAAGAACAAATGTGGCAACCTGAATACAGACCATT<br>GAGAAAAGTATGA
'tesA	ATGGCCGATACTTTGTTAATTTTGGGTGACTCTTTATCAGCCGGTTATAGAATGTCC<br>GCTAGTGCTGCATGGCCAGCATTGTTAAACGATAAATGGCAATCTAAGACTTCAGTT<br>GTCAATGCATCTATATCAGGTGACACATCACAACAAGGTTTGGCCAGATTACCAGCT<br>TTGTTAAAACAACATCAACCTAGATGGGTCTTGGTAGAATTAGGTGGTAACGATGG<br>TTTGAGAGGTTTTCAACCTCAACAAACCGAACAAACTTTGAGACAAATCTTACAAGA<br>TGTTAAGGCCGCTAATGCAGAACCATTGTTAATGCAAATTAGATTACCTGCCAACTA<br>TGGTAGAAGATACAATGAAGCATTTTCTGCAATCTATCCAAAATTGGCAAAGGAATT<br>TGATGTACCATTGTTGCCATTTTTCATGGAAGAAGTTTACTTAAAACCTCAATGGAT<br>GCAAGATGACGGTATTCATCCAAACAGAGATGCTCAACCTTTTATAGCAGACTGGAT<br>GGCCAAACAATTGCAACCATTAGTCAATCACGATTCTTGA
AAR	ATGTTCGGTTTAATAGGTCACTTAACAAGTTTAGAACAAGCCAGAGATGTCAGTAG<br>AAGAATGGGTTACGATGAATACGCAGACCAAGGTTTAGAATTTTGGTCTTCAGCCC<br>CACCTCAAATCGTAGATGAAATTACAGTTACCTCTGCTACTGGTAAAGTCATTCATG

	GTAGATACATCGAATCATGTTTCTTGCCAGAAATGTTGGCTGCAAGAAGATTCAAAACTGCAACAAGAAAGGTTTTGAATGCAATGTCCCATGCCCAAAAGCACGGTATCGATATTTCCGCATTGGGTGGTTTTACAAGTATAATCTTCGAAAACTTCGATTTGGCTAGTTTGAGACAAGTTAGAGACACTACATTGGAATTCGAAAGATTCACCACTGGTAACACCCACACTGCTTACGTCATTTGTAGACAAGTAGAAGCCGCTGCAAAAACCTTGGGTATAGATATCACACAAGCCACCGTTGCTGTTGTCGGTGCTACTGGTGACATCGGTTCCGCAGTATGCAGATGGTTGGATTTGAAATTGGGTGTTGGTGACTTAATCTTGACAGCTAGAAACCAAGAAAGATTGGATAACTTGCAAGCAGAATTAGGTAGAGGTAAAATCTTGCCATTGGAAGCCGCTTTGCCTGAAGCCGATTTTATCGTTTGGGTCGCTTCTATGCCACAAGGTGTAGTTATTGATCCAGCTACCTTAAAACAACCTTGCGTTTTGATAGACGGTGGTTATCCTAAAAATTTGGGTTCTAAGGTTCAAGGTGAAGGTATCTATGTCTTGAACGGTGGTGTCGTAGAACATTGTTTCGATATAGACTGGCAAATCATGTCAGCAGCCGAAATGGCAAGACCTGAAAGACAAATGTTTGCCTGCTTCGCTGAAGCAATGTTGTTAGAATTTGAAGGTTGGCACACTAATTTCTCTTGGGGTAGAAACCAAATTACAATAGAAAAGATGGAAGCCATCGGTGAAGCCTCTGTTAGACACGGTTTCCAACCTTTAGCCTTAGCAATCTGA
ACR1	ATGAATAAGAAGTTAGAAGCATTGTTTAGAGAAAATGTCAAGGGTAAAGTCGCTTTAATCACTGGTGCCTCCTCAGGTATCGGTTTAACTATCGCAAAAAGAATTGCTGCAGCCGGTGCCCATGTTTTGTTAGTCGCTAGAACTCAAGAAACATTGGAAGAAGTTAAGGCTGCAATCGAACAACAAGGTGGTCAAGCATCTATATTCCCATGTGATTTGACAGACATGAATGCAATAGATCAATTATCCCAACAAATCATGGCCAGTGTAGATCATGTTGACTTTTTGATTAATAACGCAGGTAGATCTATAAGAAGAGCCGTTCATGAATCATTTGATAGATTCCACGACTTCGAAAGAACAATGCAATTAAACTACTTCGGTGCTGTCAGATTGGTATTGAACTTGTTGCCTCACATGATCAAGAGAAAGAATGGTCAAATTATAAACATCTCTTCAATCGGTGTATTGGCCAACGCTACCAGATTCTCTGCTTATGTTGCATCAAAAGCCGCTTTAGATGCTTTTTCCAGATGCTTGAGTGCAGAAGTTTTGAAGCATAAGATCTCTATAACTTCAATCTATATGCCATTGGTCAGAACACCAATGATCGCACCTACCAAAATCTATAAGTACGTTCCAACATTGTCTCCTGAAGAAGCAGCCGATTTGATAGTTTATGCTATCGTCAAGAGACCTACCAGAATTGCCACTCACTTGGGTAGATTAGCTTCCATTACCTACGCAATAGCCCCAGACATAAACAACATCTTGATGTCTATTGGTTTTAATTTGTTTCCTTCCAGTACTGCTGCATTAGGTGAACAAGAAAAATTGAACTTATTACAAAGAGCCTACGCAAGATTATTCCCTGGTGAACATTGGTGA
CAR	ATGTCACCTATCACCAGAGAAGAAAGATTAGAAAGAAGAATACAAGACTTATACGCCAACGATCCTCAATTCGCCGCTGCCAAGCCAGCAACAGCCATCACCGCTGCAATTGAAAGACCAGGTTTGCCATTGCCTCAAATCATCGAAACTGTTATGACAGGTTATGCTGATAGACCTGCTTTGGCACAAAGATCAGTAGAATTTGTTACAGATGCAGGTACTGGTCATACTACATTGAGATTGTTACCACACTTCGAAACTATCTCTTACGGTGAATTATGGGACAGAATTTCTGCCTTGGCTGATGTTTTATCAACCGAACAAACTGTTAAACCTGGTGACAGAGTCTGTTTGTTGGGTTTTAATTCTGTTGACTACGCAACTATAGATATGACATTGGCCAGATTAGGTGCAGTAGCCGTTCCATTGCAAACCTCTGCCGCTATTACTCAATTACAACCAATAGTCGCTGAAACACAACCTACCATGATAGCAGCCTCTGTAGATGCTTTGGCAGACGCCACTGAATTGGCTTTATCAGGTCAAACTGCAACAAGAGTCTTAGTATTCGACCATCACAGACAAGTTGATGCCCATAGAGCTGCTGTTGAATCCGCTAGAGAAAGATTGGCAGGTAGTGCCGTTGTCGAAACTTTAGCTGAAGCAATAGCTAGAGGTGACGTTCCAAGAGGTGCTTCTGCTGGTTCTGCTCCTGGTACAGACGTCTCCGATGACAGT

	TTGGCATTGTTAATCTATACCTCTGGTTCAACTGGTGCCCCAAAAGGTGCTATGTAC<br>CCTAGAAGAAATGTTGCTACATTTTGGAGAAAGAGAACCTGGTTCGAAGGTGGTTA<br>CGAACCATCTATCACTTTGAACTTCATGCCTATGTCACATGTTATGGGTAGACAAATC<br>TTGTATGGTACTTTATGCAACGGTGGTACAGCATACTTTGTTGCCAAGTCTGACTTG<br>TCAACATTATTCGAAGATTTGGCTTTAGTCAGACCAACTGAATTAACATTCGTCCCTA<br>GAGTATGGGATATGGTTTTTGACGAATTTCAATCAGAAGTCGATAGAAGATTGGTA<br>GATGGTGCTGACAGAGTAGCTTTAGAAGCACAAGTTAAGGCAGAAATAAGAAACG<br>ATGTTTTGGGTGGTAGATATACATCTGCCTTAACCGGTTCTGCTCCAATATCAGACG<br>AAATGAAGGCTTGGGTAGAAGAATTGTTAGATATGCATTTGGTTGAAGGTTACGGT<br>TCAACTGAAGCTGGTATGATATTAATCGACGGTGCAATTAGAAGACCAGCCGTTTT<br>GGATTATAAATTGGTTGATGTCCCTGACTTGGGTTACTTTTTAACTGATAGACCACA<br>CCCTAGAGGTGAATTGTTGGTTAAGACAGATTCTTTGTTCCCAGGTTATTACCAAAG<br>AGCTGAAGTTACAGCAGATGTCTTTGATGCTGACGGTTTCTATAGAACCGGTGACAT<br>TATGGCAGAAGTCGGTCCTGAACAATTCGTATACTTAGATAGAAGAAACAACGTTTT<br>GAAATTGTCTCAGGGTGAATTTGTAACTGTTTCAAAGTTGGAAGCTGTATTCGGTGA<br>CTCTCCATTAGTTAGACAAATATATATATACGGTAATTCAGCCAGAGCTTATTTGTTA<br>GCAGTCATAGTACCAACACAAGAAGCCTTGGATGCTGTTCCTGTCGAAGAATTGAA<br>AGCCAGATTGGGTGACTCCTTGCAAGAAGTTGCAAAGGCCGCTGGTTTGCAAAGTT<br>ACGAAATCCCAAGAGATTTCATCATCGAAACCACTCCTTGGACCTTAGAAAACGGTT<br>TGTTAACTGGTATCAGAAAATTGGCTAGACCACAATTGAAAAAGCATTACGGTGAA<br>TTGTTAGAACAAATATATACTGACTTGGCCCACGGTCAAGCTGATGAATTGAGATCC<br>TTAAGACAAAGTGGTGCAGATGCCCCAGTATTAGTTACAGTCTGTAGAGCAGCCGC<br>TGCATTGTTAGGTGGTTCCGCTAGTGATGTTCAACCTGACGCACATTTTACCGATTT<br>GGGTGGTGACTCTTTGTCAGCTTTATCTTTTACAAATTTGTTGCACGAAATCTTCGAT<br>ATAGAAGTACCAGTTGGTGTCATTGTATCACCTGCTAACGATTTGCAAGCATTGGCA<br>GATTATGTTGAAGCCGCTAGAAAACCAGGTTCTTCAAGACCTACTTTTGCTTCTGTTC<br>ATGGTGCATCAAATGGTCAAGTTACAGAAGTCCACGCTGGTGACTTGTCTTTGGATA<br>AGTTCATTGATGCAGCCACTTTGGCCGAAGCTCCAAGATTACCTGCTGCAAACACTC<br>AAGTAAGAACAGTTTTGTTAACCGGTGCTACTGGTTTCTTGGGTAGATATTTGGCAT<br>TAGAATGGTTAGAAAGAATGGATTTGGTTGACGGTAAATTGATTTGCTTAGTCAGA<br>GCAAAGTCCGACACTGAAGCAAGAGCCAGATTGGATAAAACATTCGATAGTGGTG<br>ACCCAGAATTGTTAGCACATTACAGAGCTTTAGCAGGTGACCACTTGGAAGTTTTAG<br>CCGGTGACAAGGGTGAAGCTGACTTGGGTTTAGATAGACAAACATGGCAAAGATT<br>GGCTGATACCGTAGACTTAATCGTTGATCCAGCCGCTTTAGTCAACCATGTATTGCC<br>ATACTCCCAATTGTTCGGTCCTAACGCATTGGGTACTGCTGAATTGTTGAGATTGGC<br>TTTGACTTCTAAAATTAAGCCTTACTCCTACACCAGTACTATCGGTGTTGCAGATCAA<br>ATTCCACCTTCAGCCTTCACTGAAGATGCTGACATAAGAGTCATCTCCGCAACAAGA<br>GCCGTAGATGACAGTTATGCTAATGGTTACTCCAACAGTAAATGGGCAGGTGAAGT<br>TTTGTTAAGAGAAGCCCATGATTTGTGTGGTTTACCAGTTGCTGTCTTTAGATGCGA<br>CATGATTTTGGCAGATACAACCTGGGCCGGTCAATTGAACGTTCCAGATATGTTCAC<br>AAGAATGATCTTGTCCTTAGCAGCCACCGGTATAGCTCCTGGTAGTTTCTATGAATT<br>GGCTGCTGATGGTGCTAGACAAAGAGCACATTACGATGGTTTGCCAGTTGAGTTTA<br>TTGCCGAAGCTATCTCCACCTTAGGTGCTCAAAGTCAAGATGGTTTCCATACTTATCA<br>CGTAATGAATCCATACGATGACGGTATTGGTTTGGACGAATTTGTTGATTGGTTAAA<br>CGAATCTGGTTGTCCTATTCAAAGAATAGCTGATTATGGTGACTGGTTACAAAGATT

	CGAAACTGCTTTGAGAGCATTACCAGATAGACAAAGACATTCCAGTTTGTTACCTTT GTTACACAATTACAGACAACCAGAAAGACCTGTCAGAGGTTCTATTGCTCCTACAGA TAGATTCAGAGCCGCTGTACAAGAAGCAAAAATAGGTCCAGATAAGGACATCCCTC ATGTTGGTGCTCCTATTATCGTAAAGTATGTATCAGATTTGAGATTGTTGGGTTTGTT GTAA
npgA	ATGGTGCAAGACACATCAAGCGCAAGCACTTCGCCAATTTTAACAAGATGGTACAT CGACACCCGCCCTCTAACCGCCTCAACAGCAGCCCTTCCTCTCCTTGAAACCCTCCAG CCCGCTGATCAAATCTCCGTCCAAAAATACTACCATCTGAAGGATAAACACATGTCT CTCGCCTCTAATCTGCTCAAATACCTCTTCGTCCACCGAAACTGTCGCATCCCCTGGT CTTCAATCGTGATCTCTCGAACCCCAGATCCGCACAGACGACCATGCTATATTCCACC CTCAGGCTCACAGGAAGACAGCTTCAAAGACGGATATACCGGCATCAACGTTGAGT TCAACGTCAGCCACCAAGCCTCAATGGTCGCGATCGCGGGAACAGCTTTTACTCCCA ATAGTGGTGGGGACAGCAAACTCAAACCCGAAGTCGGAATTGATATTACGTGCGTA AACGAGCGGCAGGGACGGAACGGGGAAGAGCGGAGCCTGGAATCGCTACGTCAA TATATTGATATATTCTCGGAAGTGTTTTCCACTGCAGAGATGGCCAATATAAGGAGG TTAGATGGAGTCTCATCATCCTCACTGTCTGCTGATCGTCTTGTGGACTACGGGTAC AGACTCTTCTACACTTACTGGGCGCTCAAAGAGGCGTATATAAAAATGACTGGGGA GGCCCTCTTAGCACCGTGGTTACGGGAACTGGAATTCAGTAATGTCGTCGCCCCGG CCGCTGTTGCGGAGAGTGGGGATTCGGCTGGGGATTTCGGGGAGCCGTATACGGG TGTCAGGACGACTTTATATAAAAATCTCGTTGAGGATGTGAGGATTGAAGTTGCTG CTCTGGGCGGTGATTACCTATTTGCAACGGCTGCGAGGGGTGGTGGGATTGGAGCT AGTTCTAGACCAGGAGGTGGTCCAGACGGAAGTGGCATCCGAAGCCAGGATCCCT GGAGGCCTTTCAAGAAGTTAGATATAGAGCGAGATATCCAGCCCTGTGCGACTGGG GTGTGTAATTGCCTATCCTAA
SeADO	ATGCCACAATTAGAAGCCTCCTTAGAATTAGACTTTCAATCAGAATCATATAAAGAT GCTTACAGTAGAATCAACGCAATCGTCATTGAAGGTGAACAAGAAGCATTTGATAA CTACAACAGATTGGCAGAAATGTTACCAGATCAAAGAGACGAATTGCATAAATTGG CCAAGATGGAACAAAGACACATGAAAGGTTTCATGGCTTGTGGTAAAAATTTGTCC GTTACTCCTGATATGGGTTTCGCACAAAAGTTTTTCGAAAGATTGCATGAAAACTTC AAAGCTGCAGCCGCTGAGGGTAAAGTTGTCACATGTTTGTTGATCCAATCTTTGATA ATCGAATGCTTTGCTATCGCAGCCTATAATATCTACATTCCAGTCGCTGATGCATTCG CCAGAAAGATTACCGAAGGTGTAGTTAGAGACGAATATTTGCACAGAAACTTCGGT GAAGAATGGTTGAAGGCAAACTTCGATGCTTCTAAGGCAGAATTGGAAGAAGCTA ATAGACAAAACTTGCCTTTAGTCTGGTTGATGTTAAATGAAGTAGCCGATGACGCTA GAGAATTGGGTATGGAAAGAGAATCATTAGTTGAAGACTTCATGATCGCATACGGT GAAGCCTTAGAAAACATCGGTTTTACTACCAGAGAAATAATGAGAATGTCCGCATA CGGTTTGGCAGCAGTCTAA
NpADO	ATGCAACAATTAACAGACCAATCAAAGGAATTAGACTTCAAATCAGAAACTTACAAA GATGCCTACTCCAGAATCAACGCAATCGTCATTGAAGGTGAACAAGAAGCACATGA AAACTACATCACCTTGGCCCAATTATTACCAGAATCCCATGATGAATTGATCAGATT GTCTAAGATGGAATCAAGACACAAAAAGGGTTTTGAAGCCTGTGGTAGAAATTTGG CTGTTACTCCTGACTTACAATTTGCCAAAGAATTTTTCTCTGGTTTGCACCAAAACTT CCAAACTGCTGCAGCCGAGGGTAAAGTTGTCACATGTTTGTTGATCCAATCATTAAT AATCGAATGCTTTGCTATCGCTGCATATAATATCTACATTCCAGTTGCCGATGACTTC GCTAGAAAAATTACAGAAGGTGTAGTTAAGGAAGAATATTCCCATTTGAACTTTGG

	TGAAGTCTGGTTAAAAGAACACTTCGCAGAGAGTAAGGCCGAATTGGAATTAGCAA ATAGACAAAACTTGCCTATCGTCTGGAAAATGTTAAATCAAGTAGAAGGTGACGCT CATACCATGGCAATGGAAAAGGATGCTTTGGTTGAAGACTTCATGATTCAATACGG TGAAGCATTATCAAACATAGGTTTTTCTACCAGAGACATTATGAGATTGAGTGCTTA CGGTTTGATAGGTGCTTGA

References

1 Verduyn, C., Postma, E., Scheffers, W. A. & Van Dijken, J. P. Effect of benzoic acid on metabolic fluxes in yeasts: a continuous-culture study on the regulation of respiration and alcoholic fermentation. *Yeast* **8**, 501-517 (1992).

2 Khara, B. *et al.* Production of propane and other short-chain alkanes by structure-based engineering of ligand specificity in aldehyde-deformylating oxygenase. *Chembiochem : a European journal of chemical biology* **14**, 1204-1208 (2013).

3 Huh, W. K. *et al.* Global analysis of protein localization in budding yeast. *Nature* **425**, 686-691 (2003).

4 Bakker, B. M. *et al.* The mitochondrial alcohol dehydrogenase Adh3p is involved in a redox shuttle in *Saccharomyces cerevisiae*. *J. Bacteriol.* **182**, 4730-4737 (2000).

5 Kondo, T. *et al.* Genetic engineering to enhance the Ehrlich pathway and alter carbon flux for increased isobutanol production from glucose by *Saccharomyces cerevisiae*. *J. Biotechnol.* **159**, 32-37 (2012).

6 Larroy, C., Fernandez, M. R., Gonzalez, E., Pares, X. & Biosca, J. A. Characterization of the *Saccharomyces cerevisiae* YMR318C (ADH6) gene product as a broad specificity NADPH-dependent alcohol dehydrogenase: relevance in aldehyde reduction. *Biochem J.* **361**, 163-172 (2002).

7 Hansen, E. H. *et al.* De novo biosynthesis of vanillin in fission yeast (*Schizosaccharomyces pombe*) and baker's yeast (*Saccharomyces cerevisiae*). *Appl Environ Microb* **75**, 2765-2774 (2009).

8 Larroy, C., Pares, X. & Biosca, J. A. Characterization of a *Saccharomyces cerevisiae* NADP(H)-dependent alcohol dehydrogenase (ADHVII), a member of the cinnamyl alcohol dehydrogenase family. *Eur. J. Biochem.* **269**, 5738-5745 (2002).

9 Dickinson, J. R., Salgado, L. E. & Hewlins, M. J. The catabolism of amino acids to long chain and complex alcohols in *Saccharomyces cerevisiae*. *The Journal of biological chemistry* **278**, 8028-8034 (2003).

10 Toivari, M. H., Salusjarvi, L., Ruohonen, L. & Penttila, M. Endogenous xylose pathway in *Saccharomyces cerevisiae*. *Appl Environ Microb* **70**, 3681-3686 (2004).

11 Gonzalez, E. *et al.* Characterization of a (2R,3R)-2,3-butanediol dehydrogenase as the *Saccharomyces cerevisiae* YAL060W gene product - Disruption and induction of the gene. *The Journal of biological chemistry* **275**, 35876-35885 (2000).

12 Gonzalez, E. *et al.* Role of *Saccharomyces cerevisiae* oxidoreductases Bdh1p and Ara1p in the metabolism of acetoin and 2,3-butanediol. *Appl Environ Microb* **76**, 670-679 (2010).

13 Muller, M., Katzberg, M., Bertau, M. & Hummel, W. Highly efficient and stereoselective biosynthesis of (2S,5S)-hexanediol with a dehydrogenase from *Saccharomyces cerevisiae*. *Org. Biomol. Chem.* **8**, 1540-1550 (2010).

14 Jordan, D. B. *et al.* Kinetic mechanism of an aldehyde reductase of *Saccharomyces cerevisiae* that relieves toxicity of furfural and 5-hydroxymethylfurfural. *BBA-Proteins Proteom.* **1814**, 1686-1694 (2011).

15 Hwang, J. Y. *et al.* Simultaneous synthesis of 2-phenylethanol and L-homophenylalanine using aromatic transaminase with yeast ehrlich pathway. *Biotechnol. Bioeng.* **102**, 1323-1329 (2009).

16 Moon, J. & Liu, Z. L. Direct enzyme assay evidence confirms aldehyde reductase function of Ydr541cp and Ygl039wp from *Saccharomyces cerevisiae*. *Yeast* **32**, 399-407 (2015).

17 Han, G. *et al.* The *Saccharomyces cerevisiae* YBR159w gene encodes the 3-ketoreductase of the microsomal fatty acid elongase. *The Journal of biological chemistry* **277**, 35440-35449 (2002).

18 Yang, Y., Zhu, D., Piegat, T. J. & Hua, L. Enzymatic ketone reduction: mapping the substrate profile of a short-chain alcohol dehydrogenase (YMR226c) from *Saccharomyces cerevisiae*. *Tetrahedron-Asymmetr.* **18**, 1799-1803 (2007).

19 Katz, M., Hahn-Hagerdal, B. & Gorwa-Grauslund, M. F. Screening of two complementary collections of *Saccharomyces cerevisiae* to identify enzymes involved in stereo-selective reductions of specific carbonyl compounds: an alternative to protein purification. *Enzyme Microb Tech* **33**, 163-172 (2003).

20 Maruyama, R., Nishizawa, M., Itoi, Y., Ito, S. & Inoue, M. The enzymes with benzil reductase activity conserved from bacteria to mammals. *J. Biotechnol.* **94**, 157-169 (2002).

21 Athenstaedt, K. & Daum, G. 1-Acyldihydroxyacetone-phosphate reductase (Ayr1p) of the yeast *Saccharomyces cerevisiae* encoded by the open reading frame YIL124w is a major component of lipid particles. *The Journal of biological chemistry* **275**, 235-240 (2000).

22 Delneri, D., Gardner, D. C., Bruschi, C. V. & Oliver, S. G. Disruption of seven hypothetical aryl alcohol dehydrogenase genes from *Saccharomyces cerevisiae* and construction of a multiple knock-out strain. *Yeast* **15**, 1681-1689 (1999).

23 Chang, Q., Griest, T. A., Harter, T. M. & Petrash, J. M. Functional studies of aldo-keto reductases in *Saccharomyces cerevisiae*. *BBA-Mol. Cell Res.* **1773**, 321-329 (2007).

24 Petrash, J. M. *et al.* Functional genomic studies of aldo-keto reductases. *Chem.-Biol. Interact.* **130**, 673-683 (2001).

25 van Bergen, B., Strasser, R., Cyr, N., Sheppard, J. D. & Jardim, A. alpha,beta-dicarbonyl reduction by *Saccharomyces* D-arabinose dehydrogenase. *BBA-Gen. Subjects* **1760**, 1636-1645 (2006).

26 Traff, K. L., Otero Cordero, R. R., van Zyl, W. H. & Hahn-Hagerdal, B. Deletion of the GRE3 aldose reductase gene and its influence on xylose metabolism in recombinant strains of *Saccharomyces cerevisiae* expressing the xylA and XKS1 genes. *Appl Environ Microb* **67**, 5668-5674 (2001).

27 Rintala, E., Pitkanen, J. P., Vehkomaki, M. L., Penttila, M. & Ruohonen, L. The ORF YNL274c (GOR1) codes for glyoxylate reductase in *Saccharomyces cerevisiae*. *Yeast* **24**, 129-136 (2007).

28 Overkamp, K. M. *et al.* Functional analysis of structural genes for NAD(+)-dependent formate dehydrogenase in *Saccharomyces cerevisiae*. *Yeast* **19**, 509-520 (2002).

29 Buijs, N. A., Zhou, Y. J., Siewers, V. & Nielsen, J. Long-chain alkane production by the yeast *Saccharomyces cerevisiae*. *Biotechnol. Bioeng.* **112**, 1275-1279 (2015).

30 Valle-Rodriguez, J. O., Shi, S. B., Siewers, V. & Nielsen, J. Metabolic engineering of *Saccharomyces cerevisiae* for production of fatty acid ethyl esters, an advanced biofuel, by eliminating non-essential fatty acid utilization pathways. *Appl Energ* **115**, 226-232 (2014).

31 Oud, B. *et al.* An internal deletion in MTH1 enables growth on glucose of pyruvate-decarboxylase negative, non-fermentative *Saccharomyces cerevisiae*. *Microb. Cell Fact.* **11**, 131 (2012).
